# Propionate and butyrate counteract renal damage and progression to chronic kidney disease

**DOI:** 10.1093/ndt/gfae118

**Published:** 2024-05-24

**Authors:** Viviana Corte-Iglesias, Maria Laura Saiz, Ana Cristina Andrade-Lopez, Nuria Salazar, Cristian Ruiz Bernet, Cristina Martin-Martin, Jesús Martinez Borra, Juan-Jose Lozano, Ana M Aransay, Carmen Diaz-Corte, Carlos Lopez-Larrea, Beatriz Suarez-Alvarez

**Affiliations:** Translational Immunology, Health Research Institute of the Principality of Asturias (ISPA), Oviedo, Asturias, Spain; Kidney Disease Spanish Network, RICORS2040, Instituto de Salud Carlos III (ISCIII), Madrid, Spain; Department of Immunology, Hospital Universitario Central de Asturias, Oviedo, Spain; Translational Immunology, Health Research Institute of the Principality of Asturias (ISPA), Oviedo, Asturias, Spain; Kidney Disease Spanish Network, RICORS2040, Instituto de Salud Carlos III (ISCIII), Madrid, Spain; Translational Immunology, Health Research Institute of the Principality of Asturias (ISPA), Oviedo, Asturias, Spain; Department of Nephrology, Hospital Universitario San Agustin, Avilés, Spain; Department of Microbiology and Biochemistry of Dairy Products, Instituto de Productos Lácteos de Asturias (IPLA-CSIC), Villaviciosa, Spain; Diet, Human Microbiota and Health Group, Health Research Institute of the Principality of Asturias (ISPA), Oviedo, Asturias, Spain; Translational Immunology, Health Research Institute of the Principality of Asturias (ISPA), Oviedo, Asturias, Spain; Translational Immunology, Health Research Institute of the Principality of Asturias (ISPA), Oviedo, Asturias, Spain; Kidney Disease Spanish Network, RICORS2040, Instituto de Salud Carlos III (ISCIII), Madrid, Spain; Translational Immunology, Health Research Institute of the Principality of Asturias (ISPA), Oviedo, Asturias, Spain; Kidney Disease Spanish Network, RICORS2040, Instituto de Salud Carlos III (ISCIII), Madrid, Spain; Department of Immunology, Hospital Universitario Central de Asturias, Oviedo, Spain; Centro de Investigación Biomédica en Red de Enfermedades Hepáticas y Digestivas (CIBEREHD), Madrid, Spain; Centro de Investigación Biomédica en Red de Enfermedades Hepáticas y Digestivas (CIBEREHD), Madrid, Spain; CIC bioGUNE, Basque Research and Technology Alliance (BRTA), Derio, Spain; Translational Immunology, Health Research Institute of the Principality of Asturias (ISPA), Oviedo, Asturias, Spain; Department of Nephrology, Hospital Universitario Central de Asturias, Oviedo, Spain; Translational Immunology, Health Research Institute of the Principality of Asturias (ISPA), Oviedo, Asturias, Spain; Kidney Disease Spanish Network, RICORS2040, Instituto de Salud Carlos III (ISCIII), Madrid, Spain; Translational Immunology, Health Research Institute of the Principality of Asturias (ISPA), Oviedo, Asturias, Spain; Kidney Disease Spanish Network, RICORS2040, Instituto de Salud Carlos III (ISCIII), Madrid, Spain

**Keywords:** acute kidney injury, AKI-to-CKD transition, fibrosis, inflammation, short-chain fatty acids

## Abstract

**Background:**

Short-chain fatty acids (SCFAs), mainly acetate, propionate and butyrate, are produced by gut microbiota through fermentation of complex carbohydrates that cannot be digested by the human host. They affect gut health and can contribute at the distal level to the pathophysiology of several diseases, including renal pathologies.

**Methods:**

SCFA levels were measured in chronic kidney disease (CKD) patients (*n* = 54) at different stages of the disease, and associations with renal function and inflammation parameters were examined. The impact of propionate and butyrate in pathways triggered in tubular cells under inflammatory conditions was analysed using genome-wide expression assays. Finally, a pre-clinical mouse model of folic acid–induced transition from acute kidney injury to CKD was used to analyse the preventive and therapeutic potential of these microbial metabolites in the development of CKD.

**Results:**

Faecal levels of propionate and butyrate in CKD patients gradually reduce as the disease progresses, and do so in close association with established clinical parameters for serum creatinine, blood urea nitrogen and the estimated glomerular filtration rate. Propionate and butyrate jointly downregulated the expression of 103 genes related to inflammatory processes and immune system activation triggered by tumour necrosis factor-α in tubular cells*. In vivo*, the administration of propionate and butyrate, either before or soon after injury, respectively, prevented and slowed the progression of damage. This was indicated by a decrease in renal injury markers, the expression of pro-inflammatory and pro-fibrotic markers, and recovery of renal function over the long term.

**Conclusions:**

Propionate and butyrate levels are associated with a progressive loss of renal function in CKD patients. Early administration of these SCFAs prevents disease advancement in a pre-clinical model of acute renal damage, demonstrating their therapeutic potential independently of the gut microbiota.

## Graphical Abstract

**Graphical Abstract:**
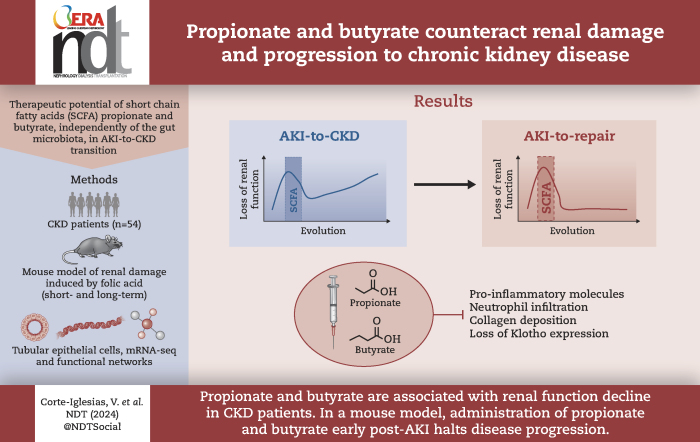

KEY LEARNING POINTS **What was known:**

- The composition of the gut microbiota can change as chronic kidney disease (CKD) develops.
- Metabolites derived from gut microbiota, such as short-chain fatty acids and uraemic toxins, may affect the physiopathology of renal damage.

**This study adds:**

- Raised faecal propionate and butyrate levels are associated with loss of renal function during CKD progression.
- Propionate and butyrate influence inflammatory and immune responses in tubular cells by altering gene transcription.
- Effects of propionate and butyrate on transition from acute injury to CKD were examined *in vivo*.

**Potential impact:**

- Propionate and butyrate levels in non-invasive faecal samples follow the progressive loss of renal function.
- Administering propionate and butyrate is potentially a therapeutic strategy for prevention and for reducing acute injury and progression towards chronic renal damage.

## INTRODUCTION

Chronic kidney disease (CKD) is a silent disorder characterized by a local inflammatory process that becomes a systemic inflammatory disease as it progresses. At the global level, around 850 million people have CKD [1] and it is predicted to become the fifth greatest global cause of death by 2040 [2]. Acute kidney injury (AKI) is a risk factor for the development of CKD, and patients who undergo AKI have an almost 9-fold higher risk of developing CKD and a 3-fold greater risk of progression to an end-stage renal disease [3]. A main goal of therapeutic research is to reduce the maladaptive repair triggered after AKI and thereby curb disease progression and its long-term consequences. Proximal tubule cells have an essential role in accumulating aberrant epigenetic and transcriptional changes that help to perpetuate the damage [4].

One approach to improving the quality of life and lifespan of CKD patients is to focus on dietary management [5]. For these patients, some dietary recommendations are clear such as reducing protein intake [6]. However, increasing dietary fibre intake has less adherence despite evidence of its multiple salutogenic effects [7, 8]. Short-chain fatty acids (SCFAs) are the main final metabolic products of carbohydrate and protein fermentation by the gut microbiota [9]. Acetate (C2), propionate (C3) and butyrate (C4) are the most abundant SCFAs, with the latter being the main energy source of colonocytes. SCFAs enter the systemic circulation through colonocytes by active transporters or passive diffusion [10]. They can act as agonists for some G protein–coupled receptors (GPRs) such as GPR41 and GPR43. Likewise, butyrate binds to GPR109A [11, 12], causing activation of several MAPK (ERK1/2, p38 and JNK) intracellular pathways. Propionate and butyrate can inhibit the activity of histone deacetylases (HDACs) leading to modification of intracellular acetylation patterns and, consequently, gene expression profiles [13]. Through their ability to reach the peripheral circulation, propionate and butyrate can exert several physiological functions in multiple organs and interfere in a variety of pathological disorders [14–18]. SCFAs are key to maintaining the integrity of the intestinal barrier and can modulate immune system activation, reduce systemic inflammation and counteract the production of uraemic toxins produced by proteolytic bacteria, among other activities [19–23].

Evidence from pre-clinical studies has shown that feeding with high levels of dietary fibre or direct administration of SCFAs can ameliorate the development of AKI [24, 25]. Two recent studies reported that manipulation of the gut microbiome helped prevent initial renal damage and its progression to CKD [26, 27]. With the aim of translating these results to a clinical setting, we investigated the dynamics of propionate and butyrate in CKD patients during disease progression and evaluated the potential use of these SCFAs as a therapeutic treatment.

## MATERIALS AND METHODS

### Patients and samples

Non-dialysed CKD patients (*n* = 54) from the Central University Hospital of Asturias (Oviedo, Spain) were enrolled in the study. CKD is defined by markers of kidney damage or decreased estimated glomerular filtration rate (eGFR) persisting for at least 3 months and classified into CKD stages based upon the eGFR levels (stage 3a: 45–59 mL/min/1.73 m^2^; stage 3b: 30–44 mL/min/1.73 m^2^; stage 4: 15–29 mL/min/1.73 m^2^; stage 5: <15 mL/min/1.73 m^2^). Exclusion criteria included patients with cancer, infectious diseases, immunosuppressive treatment or with antibiotics within 1 month before sample collection. Demographic and clinical characteristics of the CKD patients are summarized in Table 1. The study was approved by the research ethics committee of the Principality of Asturias (CEImPA, no. 86/17;87/18;106/18). Written informed consent was obtained from all participants in compliance with the Helsinki Declaration. Faecal samples were collected in sterile containers and immediately frozen at –20°C until use. Serum was harvested from blood samples and stored at –80°C.

**Table 1:** Demographic and clinical characteristics of the patients studied.

	CKD patients (*n* = 54)
Age (years; mean ± SD)	72 ± 10.32
Sex (M:F; *n*)	32:22
Weight (kg; mean ± SD)	77.5 ± 16.46
Height (cm; mean ± SD)	164 ± 9.34
BMI (kg/m^2^; mean ± SD)	30 ± 5.19
Hypertension [y/n; *n* (%)]	50(92.6)/4(7.4)
Diabetes [y/n; *n* (%)]	24 (44.4)/30 (55.5)
Etiology of CKD [*n* (%)]	
Diabetic nephropathy	20 (37.04)
Nephroangiosclerosis	16 (29.6)
Glomerular	6 (11.1)
PKD	7 (12.9)
Other	5 (9.25)
CKD stage (KDIGO) [*n* (%)]	
eGFR	
3A	7 (12.9)
3B	21 (38.8)
4	16 (29.6)
5	10 (18.5)
ACR	
A1 (<30 mg/g)	2 (3.7)
A2 (30–300 mg/g)	23 (42.6)
A3 (>300 mg/g)	29 (53.7)

BMI, body mass index; CKD, chronic kidney disease; F, female; M, male; *n*, number; PKD, polycystic kidney disease.

### SCFA quantification

For SCFA determination, 1 g of human faecal samples or 150 mg of mouse caecal samples were diluted in phosphate-buffered saline solution (PBS) at 1/10 dilution and homogenized in a LabBlender 400 stomacher (Seward Medical, London, UK). After centrifugation at 10 000 rpm for 10 min, faecal supernatants were stored at –20°C until determination. Briefly, faecal supernatants were mixed with 0.3 mL methanol, 0.05 mL internal standard solution (2-ethylbutyric 1.05 mg/mL) and 0.05 mL of 20% formic acid. This mixture was then centrifuged, and the supernatant used for SCFA quantification by gas chromatography as previously described [28]. Samples were analysed in duplicate.

### Determination of microbiota groups

The abundance of the most common intestinal microbiota groups were measured by quantitative polymerase chain reaction (PCR), using previously described primers and conditions [29]. A 7500 Fast Real-Time PCR System (Applied Biosystems, Foster City, CA, USA) was used with SYBR Green chemistry (Applied Biosystems). Bacterial levels were determined by including standard curves derived from pure cultures of appropriate strains. Results were expressed as the log_10_ of the cell count per gram of faeces.

### Cytokine detection

Concentrations of a panel of inflammatory mediators (cytokines and chemokines) were measured in serum samples using a Luminex custom multiplexed assay, in accordance with the manufacturer's protocol. After cytokine labelling, proteins were detected in a Luminex^®^ 100/200 System using xPONENT^®^ software (Luminex, Austin, TX, USA).

### Cell culture and treatments

Human proximal tubular epithelial cells (TECs) (HK-2 cell line; CRL-2190; ATCC, Manassas, VA, USA) were maintained in RPMI 1640 medium (Gibco, Carlsbad, CA, USA) supplemented with 10% foetal bovine serum, 1% penicillin/streptomycin, 1% insulin transferrin selenite (Gibco) and 5 ng/mL hydrocortisone (Sigma–Aldrich, St Louis, MO, USA). Cells (0.25 × 10^6^ cells/mL) were treated with acetic (25 mM), propionic (15 mM) and butyric (3 mM) acids (all from Sigma–Aldrich) for 24 h before or after induction with tumour necrosis factor (TNF-α) (5 ng/mL; 3 h; Prepotech Inc., London, UK).

### RNA sequencing and gene expression analysis

RNA was isolated from HK-2 cells with a PureLink™ RNA Mini Kit (Invitrogen, Carlsbad, CA, USA). The integrity of the RNA was analysed in RNA 6000 Nano Chips with a Bioanalyzer 2100 (Agilent Technologies, Santa Clara, CA, USA) and quantified using a fluorometric Qubit RNA HS assay kit (Invitrogen). Briefly, 1000 ng of total RNA were used to prepare sequencing libraries following the TruSeq Stranded mRNA Sample Preparation Guide (Part #15031058 Rev. E) and using the TruSeq Stranded mRNA Library Prep kit (Illumina Inc., San Diego, CA, USA, Cat. #20020294) and TruSeq RNA Single Indexes (Illumina Inc., Cat. #20020492 and 20020493). Sequencing was carried out using a HiSeq 2500 platform (Illumina Inc.). Files of data generated by the sequencing were aligned against the human genome (Homo_sapiens.GRCh38.94.gtf) using the STAR program [30], and the genes and transcripts were analysed with the RSEM program [31] using GENCODE v26 [32]. Expression data were processed using limma and edgeR packages [33]. Briefly, we previously eliminate genes with a geometric mean expected value lower than 1, and we used TMM method and limma-voom transformation to normalize the non-biological variability. Genes significantly differentially expressed between groups were assessed using moderated t-statistics and given the criteria of >1.5-fold change and an adjusted *P* < .05 were fulfilled. Genes were compared among groups using Venn diagrams [34]. Gene Ontology enrichment analysis was conducted using the DAVID web-based tool, DAVID Knowledgebase (v2023q4) [35, 36]. Biological processes were determined using *Homo sapiens* whole genome as gene background. Raw mRNAseq data have been deposited in the NCBI Gene Expression Omnibus under accession number GEO:GSE221506.

### Gene expression analysis

Total RNA from HK-2 cells or frozen kidneys was isolated using a GeneMATRIX Universal RNA purification kit (EURx, Gdansk, Poland) following the manufacturer's instructions. Purified RNA (1 µg) was reverse-transcribed to cDNA using a high-capacity cDNA reverse-transcription kit (Applied Biosystems). The reverse-transcription PCR (RT-PCR) using TB Green Premix Ex TaqII (Takara Bio Inc., Kusatsu, Japan) or TaqMan Gene Expression Master Mix (Applied Biosystems) followed by analysis with a StepOnePlus^TM^ Real-Time PCR System (Applied Biosystems) using the 2-ΔCt method were used to quantify gene expression. The primers and TaqMan assays used are listed in Supplementary data, Table S1.

### Mice and treatments

Male C57BL/6N mice (8–10 weeks old) were purchased from Janvier Labs (Le Genest-Saint-Isle, France) and adapted for 1 week before the study. All procedures were approved by the Animal Experimentation Committee of the Universidad de Oviedo (Spain) and performed in accordance with the European and Spanish legislative and regulatory guidelines (European Convention ETS 123, Spanish Law 6/2013, and R.D. 53). Renal damage was induced by intraperitoneal (ip) injection of a single dose of 250 mg/kg of folic acid (Sigma–Aldrich, St Louis, MO, USA) using 0.3 M NaHCO_3_ as the vehicle. Sodium butyrate (500 mg/kg) or sodium propionate (200 mg/kg) were dissolved in PBS buffer (pH = 7.4) and administered by ip injection or by oral gavage according to the indicated schemes. For the analysis in the absence of gut microbiota, mice received an antibiotic cocktail containing 1 g/L ampicillin, 0.5 g/L neomycin, 0.5 g/L vancomycin and 0.01 g/L amphotericin B in drinking water for 2 weeks, with replacement every 2 days. Mice were euthanized at short-term (24 h) or long-term (45 days) periods, and kidney and blood samples were harvested for further analysis.

### Renal function assessment

The GFR was measured by transcutaneous determination using fluorescein–isothiocyanate-labelled sinistrin (FITC-sinistrin, 150 mg/kg) and a miniaturized imager device (Medibeacon GmbH, Germany) as previously described [37]. After recording for 1.5 h, the device was recovered, and the data were analysed using MPD Studio 3 software (Medibeacon GmbH). The GFR (µL/min) was calculated from the decrease in fluorescence intensity over time (i.e. plasma half-life of FITC-sinistrin) using a two-compartment model, body weight of the mouse and an empirical conversion factor. Serum urea and creatinine were determined using the blood urea nitrogen (BUN) colorimetric detection kit (K024-H5, Arbor Assays, MI, USA) and QuantiChrom^TM^ Creatinine Assay kit (DICT-500, BioAssay Systems), respectively, following the manufacturer's instructions.

### Renal histology

Kidneys were fixed in 4% formaldehyde for 24 h and embedded in paraffin. Slices (5 μm) were stained with periodic acid–Schiff (PAS) staining for pathological injury. A minimum of 10 fields for each kidney slide were analysed. Evidence for cell injury (loss of brush border, vacuolization), cell desquamation, tubular dilation, cast formation and microhematuria were scored on a semiquantitative scale from 0 to 3. Results from each of the six items were summed to yield the histological AKI score, which therefore had a maximum value of 18. The scores were analysed by a pathologist in a blinded manner. Masson staining using Masson's Trichrome Staining Kit following manufacturer's instructions (Bio-Optica, Milan, Italy) was used to estimate renal fibrosis, and collagen content was quantified by analysing the percentage of stained area in randomly selected fields (×40) using ImageJ 1.53k software [National Institutes of Health (NIH), Bethesda, MD, USA]. Data are expressed as positively stained area as a proportion of the total area analysed. All samples were examined in a blind manner. Images were acquired with an optical microscope (DM2500; Leica) equipped with a CCD camera (DFC420; Leica) with Leica Application Suite software (version 4.3.0) and the PathoZoom^®^ Digital Lab system (Smart In Media AG, Cologne, Germany).

### Western blotting

Proteins were extracted from kidneys into RIPA lysis buffer supplemented with a protease and phosphatase inhibitor cocktail (Merck Millipore, Burlington, MA, USA) by mechanical disruption using an electric tissue homogenizer (T-10 basic ULTRA-TURRAX, IKA, Darmstadt, Germany). After SDS-PAGE, proteins were detected by western blot analysis using the following primary antibodies: NGAL (Sc-50350, 1:500; Santa Cruz Biotechnology, Santa Cruz, CA, USA), KIM-1 (AF1817, 1:1000; R&D Systems), Col1a1 (ab279711; Abcam, Cambridge, UK), α-SMA (ab242395; Abcam), fibronectin (ab2413; Abcam) and Vinculin (ab155120; Abcam) followed by incubation with horseradish peroxidase–conjugated IgG secondary antibody for 1 h. Images were captured using an ImageLab system (Bio-Rad, Hercules, CA, USA) and quantified with ImageJ version 1.53k software (NIH).

### Flow cytometry

Kidneys were minced into small pieces and digested using Liberase TM (0.2 mg/mL, Roche) and DNAse I (40 µg/mL, Roche) for 30 min at 37°C with shaking, then blocked with 0.2% PBS–0.05 mM EDTA. Samples were subsequently disrupted by passing through a 70-μm cell strainer to obtain a cell suspension that was incubated with anti-mouse FcRII/III (clone 2.4G2, TONBO^TM^ Biosciences; Cytek Biosciences, CA, USA) and Zombie Red Fixable Viability Dye (BioLegend, MA, USA) for 20 min at 4°C. The following antibodies were then used: CD45-PeCy7, CD11b-FITC, Ly6G-PE, F4/80-APC and Ly6C-PerCP (all from TONBO Biosciences), for 30 min at 4°C. Absolute cell numbers were determined using TruCount Tubes (BD Biosciences, NJ, USA). Cell samples were acquired in a spectral flow cytometer (Northern Lights 3L, Cytek Biosciences) and analysed with FlowJo software (v10.8.1, BD Biosciences).

### Statistical analysis

Data were summarized as the mean and standard deviation (SD). Characteristics of groups were compared using Fisher’s exact test for categorical variables, and the Wilcoxon paired-samples test, or the Mann–Whitney U test for unpaired samples. Correlations were calculated using non-parametric Spearman correlation coefficients. Statistical analyses were performed with IBM SPSS Statistics for Windows version 27.0 (IBM Corp., Armonk, NY, USA) and GraphPad-Prism v7 (GraphPad Software, San Diego, CA, USA). Statistical significance was concluded for values of *P* < .05. Statistical details of the experiments and significance are noted in the respective figures and legends.

## RESULTS

### SCFAs are reduced during CKD progression independently of the gut microbiota profile

**Figure 1:**
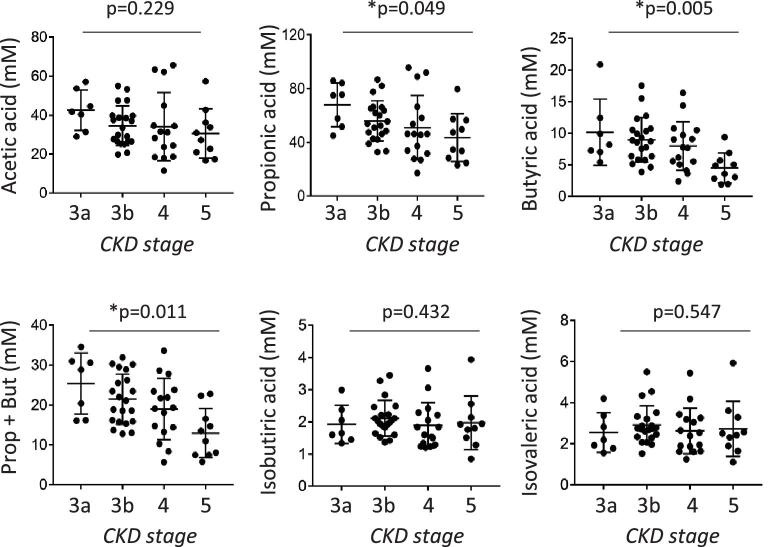
Faecal levels of propionic (Prop) and butyric (But) acids are decreased in CKD patients. SCFAs (acetic, Prop, But, Prop + But) and BCFAs (isobutyric and isovaleric acids) were quantified in faecal samples from patients at KDIGO stages 3a (*n* = 7), 3b (*n* = 21), 4 (*n* = 16) and 5 (*n* = 10). Values for each patient are shown as absolute numbers. Stage results are represented as the mean ± SD. Groups were compared using the Kruskal–Wallis test; values of *P* < .05 were considered significant (*).

The primary aim of this study was to assess the main SCFAs, i.e. acetic, propionic and butyric acids, in a cohort of CKD patients at different stages of the disease. Patient characteristics are summarized in Table 1. There were reductions in all SCFAs with disease progression; these were statistically significant for propionic (*P* = .049) and butyric (*P* = .005) acids and the sum of the two (Fig. 1). A good correlation (r = 0.5851; *P* < .0001) between these two SCFAs implies coordinated production (Supplementary data, Fig. S1). We also determined the levels of two branched short-chain fatty acids (BCFAs), isobutyric and isovaleric acid, which are produced by gut microbiota during the fermentation of branched-chain amino acids and related to nutritional status [38]. No changes in BCFA levels were detected (Fig. 1). Quantifying the most representative gut microbiota members (*Akkermansia, Bacteroides, Bifidobacterium, Clostridium* cluster XIVa, *Lactobacillus* group, *Clostridium* cluster IV and Enterobacteriaceae) revealed no significant changes associated with the progression of the disease or with the levels of propionic and butyric acids (Supplementary data, Fig. S2A and B).

### Propionic and butyric acid levels track the loss of renal function

**Figure 2:**
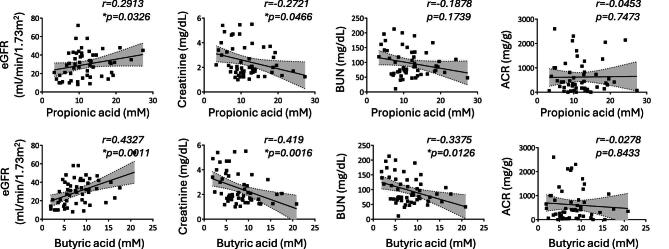
Correlation between propionic and butyric acids levels and clinical parameters in CKD patients. The associations between the absolute concentration of propionic or butyric acid and the levels of eGFR, creatinine, BUN and ACR were determined in all CKD patients (*n* = 54) at different stages of their disease. Spearman correlation coefficients were calculated; values of *P* < .05 were considered statistically significant (*).

Next, we determined whether propionic and butyric acid levels were bidirectionally associated with the progression of kidney damage. BUN, eGFR and creatinine levels were strongly associated with CKD stages, but not with the albumin-to-creatinine ratio (ACR) (Table 2). Propionic and butyric acid levels, and the sum of the two, were positively correlated with eGFR and inversely correlated with serum creatinine and BUN (not statistically significantly for propionate) levels (Fig. 2 and Supplementary data, Fig. S3). No association was observed with ACR levels (Fig. 2 and Supplementary data, Fig. S3).

**Table 2:** Clinical and immunological parameters of CKD patients by KDIGO stage.

	CKD stage				
	3a (*n* = 7)	3b (*n* = 21)	4 (*n* = 16)	5 (*n* = 10)	*P*
Age (years; mean ± SD)	62 ± 5.52	73 ± 7.45	69.5 ± 13.19	75 ± 10.33	**.017**
Sex (*n*)					
Male	4	13	9	6	.987
Female	3	8	7	4	
Weight (kg; mean ± SD)	87 ± 22.15	83 ± 12.61	76 ± 18.22	77.5 ± 16.33	.307
Height (cm; mean ± SD)	163 ± 11.27	165 ± 7.76	161.5 ± 12.07	163.5 ± 7.64	.954
BMI (kg/m^2^; mean ± SD)	33 ± 8.11	30.4 ± 3.29	28.85 ± 5.75	27 ± 5.35	.56
eGFR (mL/min/1.73 m^2^; mean ± SD)	48 ± 9.36	36 ± 4.83	21 ± 4.67	10.5 ± 1.96	**<.0001**
Creatinine (mg/dL; mean ± SD)	1.24 ± 0.159	1.7 ± 0.28	2.75 ± 0.73	4.7 ± 0.73	**<.0001**
BUN (mg/dL; mean ± SD)	48 ± 11.01	73 ± 18.09	110.5 ± 26.44	170.5 ± 57.19	**<.0001**
ACR (mg/g; mean ± SD)	210 ± 152.4	314 ± 768.2	394 ± 686.8	820 ± 598.6	.097
Total protein (g/L; mean ± SD)	70 ± 4.01	69 ± 6.13	71 ± 4.68	72 ± 6.32	.732
Serum albumin (g/L; mean ± SD)	45 ± 2.76	42 ± 2.73	43 ± 6.34	43.5 ± 2.21	.481
Serum calcidiol (ng/mL; mean ± SD)	19 ± 3.97	19.7 ± 12.3	18.6 ± 17.53	23 ± 12.12	.986
Fe (µg/dL; mean ± SD)	62.5 ± 22.51	67 ± 23	61.5 ± 19.77	66 ± 15.93	.995
Ferritin (ng/mL; mean ± SD)	119 ± 207.48	80 ± 71.81	109.5 ± 122.5	143.5 ± 192	.138
Transferrin (mg/dL; mean ± SD)	242 ± 74.13	246 ± 6.046	223 ± 63.07	212 ± 41.13	.181
Etiology of CKD (*n*)					
Diabetic nephropathy	1	9	6	4	.421
Nephroangiosclerosis	5	5	3	3	
Glomerular	0	4	1	1	
PKD	0	2	4	1	
Other	1	1	2	1	
TNF-α (pg/mL; mean ± SD)	76.6 ± 9.56	82.7 ± 22.69	98.9 ± 14.99	114.8 ± 30.8	**.0032**
CXCL8 (IL8) (pg/mL; mean ± SD)	85.6 ± 38.03	116.6 ± 64.24	146.6 ± 104.2	146.8 ± 30.83	**.0420**
CCL2 (ng/mL; mean ± SD)	1.40 ± 0.32	1.44 ± 0.85	1.48 ± 0.43	1.44 ± 0.78	.7331
GM-CSF (pg/mL; mean ± SD)	45.7 ± 20.12	51 ± 14.7	51.0 ± 12.31	51 ± 24.2	.2669
CXCL2 (ng/mL; mean ± SD)	2.40 ± 0.61	2.66 ± 0.49	2.03 ± 0.94	3.27 ± 1.03	.0669
CCL20 (ng/mL; mean ± SD)	0.16 ± 0.04	0.24 ± 0.25	0.29 ± 0.10	0.20 ± 0.44	.2583

Values in bold are statistically significant (*P* < .05).

*n*, number; CKD, chronic kidney disease; PKD, polycystic kidney disease; BMI, body mass index.

Serum levels of several pro-inflammatory cytokines and chemokines (TNF-α, CXCL8/IL8, CCL2, GM-CSF, CXCL2 and CCL20) were also quantified (Table 2). Significant increases in TNF-α and CXCL8/IL8 levels were detected during progression of the disease (Table 2 and Supplementary data, Fig. S4A). On the other hand, no association with SCFA levels was detected (Supplementary data, Fig. S4B).

### Propionate and butyrate modify the transcriptional profile of TECs

**Figure 3:**
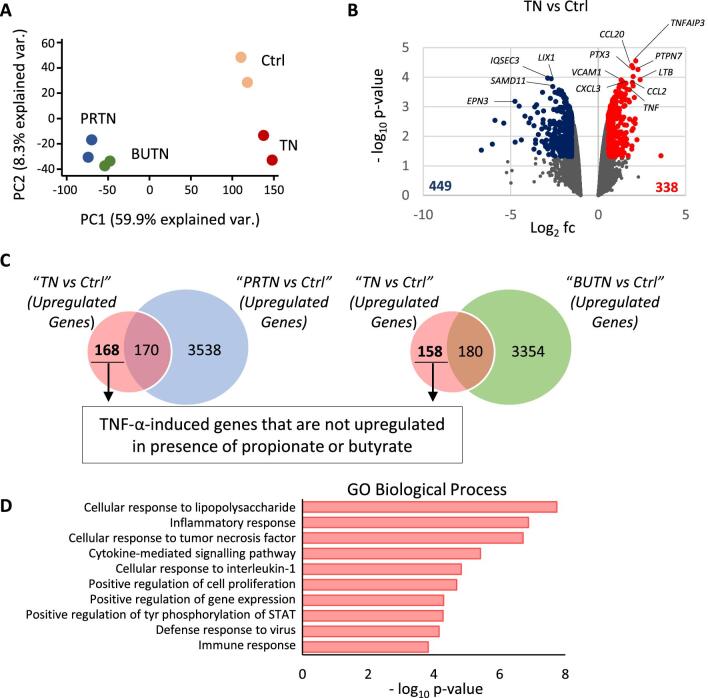
Propionate (Prop) and butyrate (But) modulate transcriptional profiles induced by TNF-α (TN) in TECs. HK2 cells were treated with Prop (15 mM) or But (3 mM) for 24 h before induction with TNF-α (5 ng/mL) for 3 h. RNA sequencing was conducted in duplicate under basal conditions (control group, Ctrl), after TNF-α induction, and in samples pre-treated with Prop (PRTN) and But (BUTN) before TNF-α induction. (**A**) Principal component (PC) analysis of gene expression profiles (two biological replicates) of the sequenced samples. (**B**) Volcano plots of the TN vs Ctrl comparison showing the differentially expressed genes (DEGs). Downregulated genes (*n* = 448) are shown in blue and upregulated genes (*n* = 338) in red if they fulfil the criteria of >1.5-fold change (fc) and adjusted *P*-value <.05. Some of the most significant DEGs are indicated. (**C**) Venn diagrams showing the upregulated genes for the two comparisons [TN vs Ctrl and PRTN vs Ctrl or BUTN vs Ctrl; >1.5-fold change (fc) and adjusted *P* < .05] to identify the genes induced by TNF-α that are not increased in the presence of Prop or But. (**D**) Histogram of the 10 most significant biological process, according to the Gene Ontology (GO) functional enrichment analysis with the DAVID database, of the 147 common genes induced by TNF-α (TN) and that are not increased in the presence of Prop (PRTN) or But (BUTN).

The human TEC line HK2 was used to determine the effect of these SCFAs in a damage condition characterized by renal inflammation. Transcriptional profiles were analysed by mRNA sequencing after treatment with TNF-α in the absence or presence of sodium propionate or butyrate. Two-dimensional principal component analysis revealed that, in the presence of TNF-α, there were some differences in the gene transcriptional profile compared with controls (Fig. 3A). However, the pre-treatment with propionate and butyrate greatly modified the transcription patterns, over and above the differences induced by TNF-α (Fig. 3A).

Initially, 338 genes were identified as being TNF-α-induced (Fig. 3B and Supplementary data, Table S2), of which 168 and 158 genes were not increased in the presence of propionate and butyrate, respectively (Fig. 3C and Supplementary data, Table S2). Gene Ontology analysis showed that both SCFAs downregulated most of the biological processes induced by TNF-α (Fig. 3D and Supplementary data, Table S2). Moreover, 87.5% (147/168) and 93% (147/158) of the genes modulated by propionate and butyrate, respectively, are common, and only a small group of genes were specific to each SCFA (Supplementary data, Fig. S5A and Table S2). These results were confirmed by RT-PCR, showing that genes involved in inflammation (*CCL2, CSF1, CXCL2, CXCL3, CXCL8, IL6, TNIP1, NFKB1, CD40* and *VCAM1*) and immune response (*CSF2, LIF* and *LTB*) were downregulated by SCFAs under inflammatory conditions and in a dose-dependent manner (Supplementary data, Fig. S5B).

We also wanted to examine the pathways modulated by propionate and butyrate independently of the inflammatory conditions. To this end, we compared the conditions of pre-treatment with both SCFAs with respect to cells induced with TNF-α (Supplementary data, Fig. S6A and B). Gene Ontology analysis revealed that propionate and butyrate induced the expression of genes related to cell adhesion and signalling pathways, and importantly, reduced the expression of many genes associated with key processes in acute renal damage, such as the cell cycle, oxidative stress and cell death (Supplementary data, Fig. S6C and D). Moreover, several pathways associated with chromatin remodelling were modified, especially those involved in changes in histone acetylation (Supplementary data, Fig. S6D).

### Therapeutic effect of propionate and butyrate in mitigating inflammation

**Figure 4:**
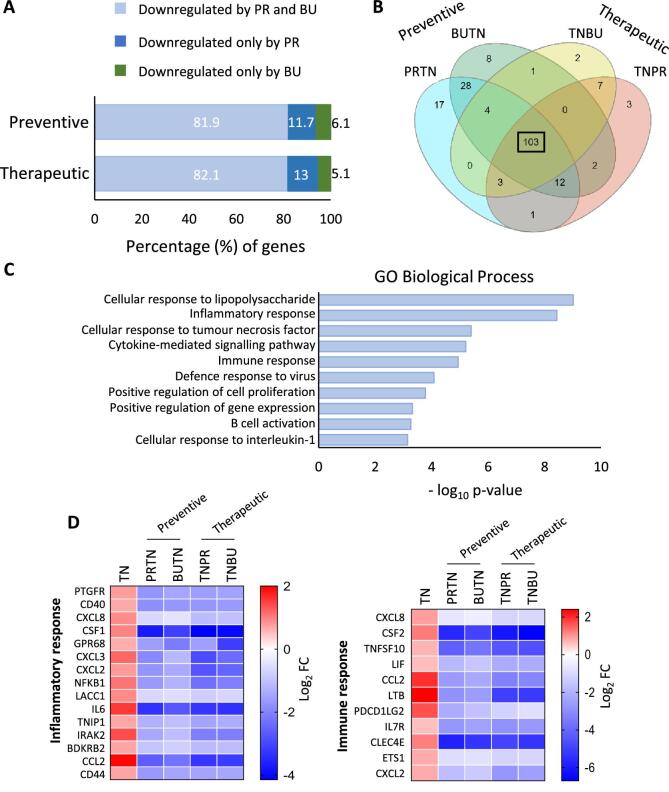
Similar preventive and therapeutic effects of propionate (Prop) and butyrate (But) treatment under inflammatory conditions. HK2 cells were treated with Prop (15 mM) or But (3 mM) for 24 h, before (preventive effect, PRTN or BUTN) or after (therapeutic effect, TNPR or TNBU) inflammation induction with TNF-α (5 ng/mL) for 3 h. Whole-genome RNA sequencing was performed. (**A**) Histogram showing the percentage of genes downregulated by Prop, But or both in preventive and therapeutic treatments. (**B**) Venn diagram showing the comparisons among genes downregulated by Prop or But under preventive and therapeutic conditions. (**C**) Histograms of the ten most significant biological processes determined by Gene Ontology (GO) functional enrichment analysis of the 103 common genes regulated by propionate and butyrate, in both preventive and therapeutic treatments. (**D**) Heatmaps of the ‘inflammatory response’ and ‘immune response’ biological processes showing the upregulated genes (red) with TNF-α (TN) and downregulated genes (blue) by Prop and But (PRTN, BUTN, TNPR, TNBU). Data are shown as the log2 fold change (FC) of each experimental condition compared with control (untreated cells).

To determine whether propionate and butyrate could be of therapeutic value, SCFAs were added after induction with TNF-α, and the results compared. More than 80% of genes were downmodulated by both metabolites regardless of the timing of administration (Fig. 4A). Comparative analysis identified 103 genes (Fig. 4B and Supplementary data, Table S3) associated at the Gene Ontology level, mainly with the inflammatory and immune responses (Fig. 4C), where genes involved in the differentiation and recruitment of immune cells, such as *CSF1* (*M-CSF*), *CSF2* (*GM-CSF*), *CCL2, CXCL8* (*IL-8*), *CXCL2* (*MIP2a*) and *CXCL3* (*MIP2b*), signalling mediated by NF-κB (*NFKB1, TNIP1, IRAK2*) and cytokine expression (*CD40, IL6, TNFSF10, LIF, IL7R, LTB*) were significantly downregulated by SCFAs (Fig. 4D).

### *In vivo* propionate and butyrate administration impairs progression to CKD

To determine whether propionate and butyrate could help counteract progression towards chronic damage, a pre-clinical model of nephropathy induced by folic acid was developed and long-term (45 days) renal damage and function were analysed. The SCFAs administration regimen included five doses given early at around the time of damage, in contrast to a previous study in which SCFAs or high-fibre diets were maintained throughout the experiment [26]. Here, SCFAs were administered 24 h before the damage and daily until the third day thereafter (Supplementary data, Fig. S7A). Results showed that eGFR and BUN levels assayed long term were preserved in the groups treated with propionate and butyrate (Supplementary data, Fig. 7B and C). The kidneys had a similar appearance to those of the control group, with no evidence of renal shrinkage with uneven surfaces, as was characteristic of the kidneys from the renal-damage group (FAN mice group) (Supplementary data, Fig. 7D). Moreover, reduced expression of the kidney damage markers KIM-1 and NGAL (Supplementary data, Fig. S7E) and a clear diminution of collagen deposits in the interstitial space (Supplementary data, Fig. S7F and G) in the treated group indicated the potential of SCFAs for preventing progression towards chronic renal damage.

**Figure 5:**
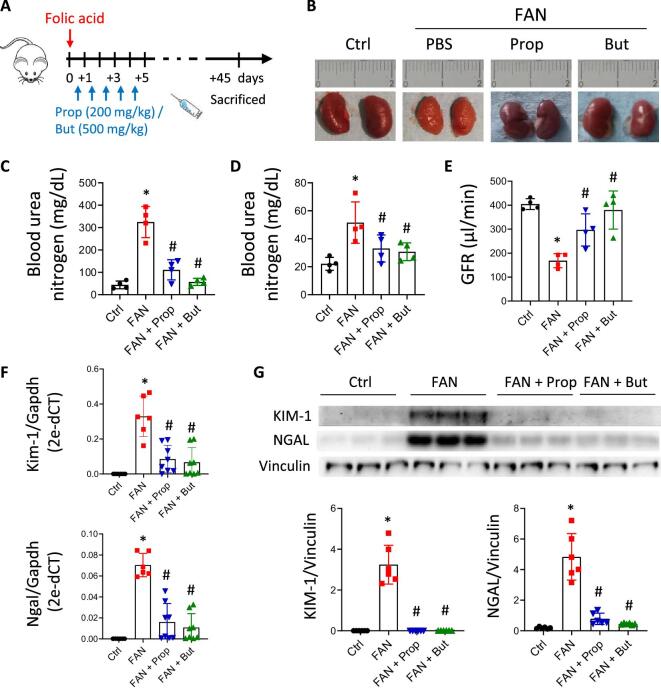
Administration of propionate (Prop) and butyrate (But) after acute damage restores renal function and injury markers. (**A**) Graphic representation of the AKI-to-CKD transition mouse model induced by high doses of folic acid (250 mg/kg) and ip administration of Prop (200 mg/kg) and But (500 mg/kg), beginning 3 h after damage and continuing for 5 consecutive days. Renal function and kidneys were analysed at Day +45 in the following groups: control (Ctrl; *n* = 6), folic acid nephropathy (FAN; *n* = 6), FAN pre-treated with Prop (Prop + FAN; *n* = 8) and FAN pre-treated with But (But + FAN; *n* = 8). (**B**) Representative images of kidneys from the different groups. Determinations of the BUN at early time, 48 h (**C**), and at long term, 45 days (**D**), and measurement of the transcutaneous GFR at Day +45 (**E**) (*n* = 4 mice per group). Expression of the renal damage markers KIM-1 and NGAL from each group by RT-PCR (**F**) and western blotting (**G**) analyses (*n* = 6–8 mice per group). *Gapdh* and vinculin were used as endogenous controls for each respective assay. Data are shown as the mean ± SD. *P < .05 vs Ctrl; ^#^*P* < .05 vs FAN as determined by the Mann–Whitney test.

**Figure 6:**
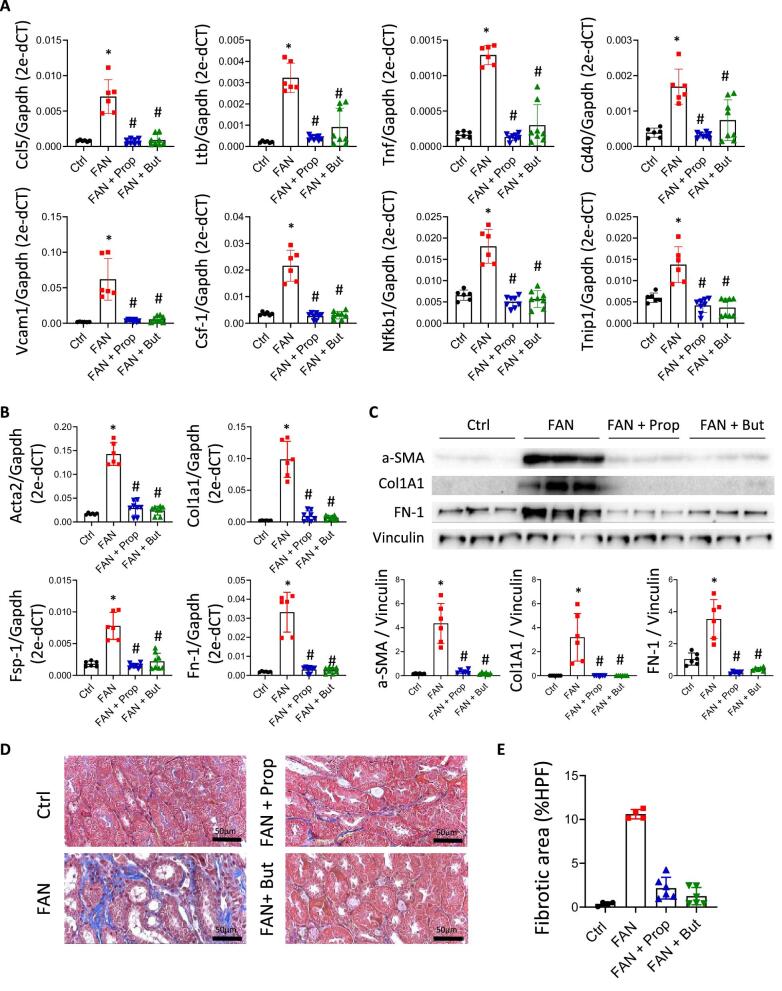
Early administration of propionate (Prop) and butyrate (But) after renal damage limits progression to CKD. Mice were treated with high doses of folic acid (250 mg/kg) and 3 h after damage Prop (200 mg/kg) and But (500 mg/kg) were administered over the next 5 days. Kidneys were examined at Day +45 in the following groups: control (Ctrl; *n* = 6), folic acid nephropathy (FAN; *n* = 6), FAN pre-treated with Prop (Prop + FAN; *n* = 8) and FAN pre-treated with But (But + FAN; *n* = 8). (**A**) Transcriptional expression of pro-inflammatory molecules in kidney tissue analysed by RT-PCR (*n* = 6-8 mice per group). Expression of fibrosis markers by RT-PCR (**B**) and western blot (**C**) in kidney tissue (*n* = 6-8 mice per group). *Gapdh* and vinculin were used as endogenous controls for the respective assays. (**D, E**) Representative images (40× magnification) of Masson's trichrome staining at Day +45 in the different groups and the corresponding quantification of their fibrotic areas (%HPF, high-power field). Scale bar is indicated in the images. *P < .05 vs Ctrl; ^#^*P* < .05 vs FAN as determined by the Mann–Whitney test. Data are shown as the mean ± SD.

To determine the impact of propionate and butyrate once damage had been initiated, SCFAs were administered 3 h after folic acid–induced damage and daily until five doses had been given (Fig. 5A). As before, the SCFA-treated group had kidneys with a size and form similar to those of the control group (Fig. 5B). Renal function, as determined by BUN levels, was partially recovered soon (48 h) after damage (Fig. 5C) and low levels were maintained over the long term (45days), consistent with higher eGFR in the treatment groups (Fig. 5D and E). The renal injury markers KIM-1 and NGAL, assayed at transcriptional and protein levels, were significantly reduced after SCFA treatment (Fig. 5F and G). Additionally, the expression of inflammatory markers, such as *Ccl5, Ltb, Tnf, Cd40, Vcam1* and *Csf1* and those related to NF-κB signalling, such as *Nfkb1* and *Tnip1*, were less strongly induced in the presence of propionate and butyrate (Fig. 6A). In other words, it is associated with less expression of extracellular matrix components (*Acta2, Col1a1, Aifm2, Fn1*) (Fig. 6B and C) and reduction in renal interstitial fibrosis as assayed by collagen staining (Fig. 6D and E).

### Early intervention with SCFAs after damage is key to inhibiting transition to CKD

**Figure 7:**
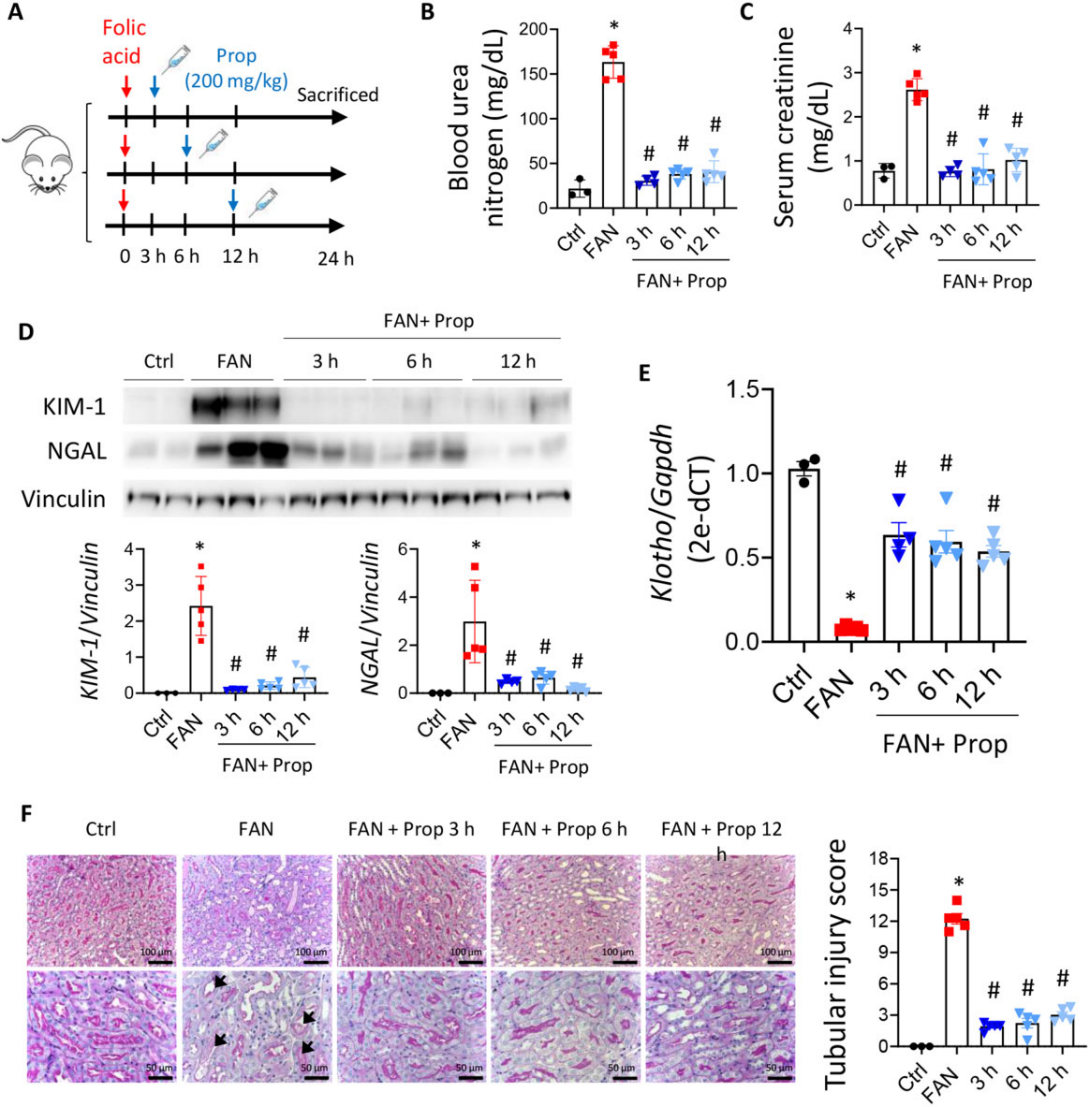
Therapeutic window of propionate (Prop) to revert initial renal damage. (**A**) Graphic representation of the renal damage mouse model induced by high doses of folic acid (250 mg/kg). Prop (200 mg/kg) was ip administered 3 h, 6 h and 12 h after folic acid, and parameters were analysed 24 h after initial damage. Groups: control (Ctrl; *n* = 3), folic acid nephropathy (FAN; *n* = 5), FAN + Prop 3h (*n* = 4), FAN + Prop 6h (*n* = 5) and FAN + Prop 12h (*n* = 5). Quantification of the serum levels of BUN (**B**) and creatinine (**C**). (**D**) Determination of protein levels of the renal injury markers KIM-1 and NGAL in kidney tissue by western blotting. Vinculin was used as an endogenous control. (**E**) Quantification of transcriptional levels of klotho (*Kl*). *Gapdh* was used as an endogenous control. (**F**) Representative images of periodic acid–Schiff-stained sections (upper panel = 20×; bottom panel = 40× magnification) and quantification of the damage in the different groups. Scale bars are indicated in the images. Arrows indicate presence of tubular casts, cell desquamation, microhematuria and loss of brush border. Data are shown as the mean ± SD. *P < .05 vs Ctrl; ^#^*P* < .05 vs FAN as determined by the Mann–Whitney test.

The time after the initial injury that SCFA administration continued to be effective was determined (Fig. 7A). Only propionate was assayed due to the similar effect of the two SCFAs. Results showed that administering propionate up to 12 h after damage was able to maintain reduced levels of BUN and serum creatinine (Fig. 7B and C) and of the renal damage markers, KIM-1 and NGAL (Fig. 7D). Partial recovery of klotho expression was observed after propionate treatment (Fig. 7E). Histological analysis showed less renal damage in all treatment groups (Fig. 7F).

**Figure 8:**
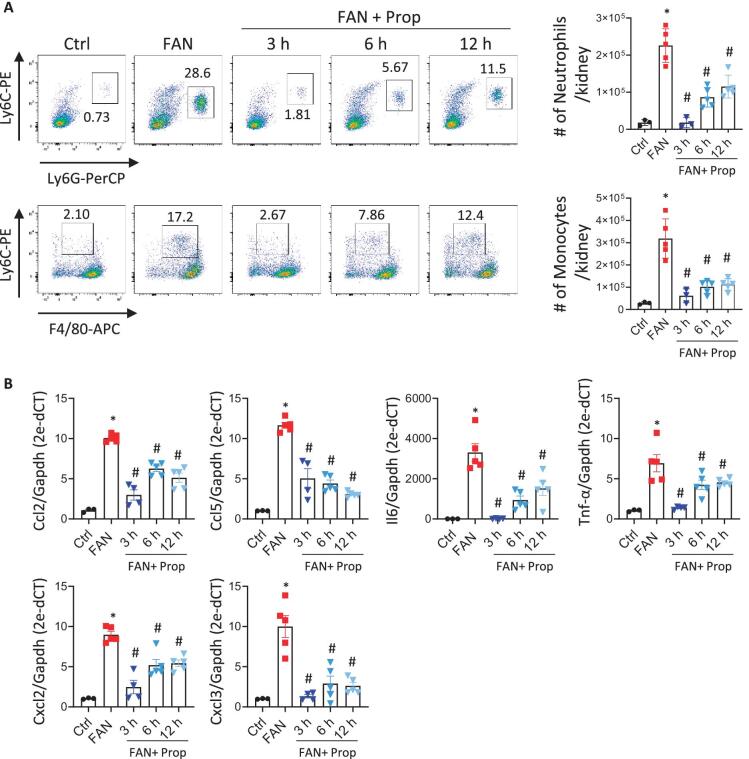
Propionate (Prop) decreases immune cell infiltration and inflammation shortly after kidney damage. (**A**) Determination by flow cytometry analysis of the number of neutrophils and monocytes infiltrating the kidney in the mouse groups: control (Ctrl; *n* = 3), folic acid nephropathy (FAN; *n* = 5), FAN + Prop 3h (*n* = 4), FAN + Prop 6h (*n* = 5) and FAN + Prop 12h (*n* = 5). The left panels show representative dot plots of each group, and numbers indicate the percentage of neutrophils or monocytes from parental cells. Complete gating strategy is shown in Supplementary data, Fig. S9. The right histograms represent the quantification of the total cell number, neutrophils or monocytes, per kidney. (**B**) Expression of pro-inflammatory molecules in kidney tissue analysed by RT-PCR in the different groups. *Gapdh* was used as an endogenous control (*n* = 3–5 mice per group). Data are shown as the mean ± SD. *P < .05 vs Ctrl; ^#^*P* < .05 vs FAN as determined by the Mann–Whitney test.

To verify the observed *in vitro* changes related to weaker inflammatory and immune responses by SCFAs in this model, the immune cells infiltrating the kidney were analysed by flow cytometry (Supplementary data, Fig. S8). A significant increase of leukocytes, mainly neutrophils and monocytes, was observed after damage and infiltration was drastically reduced following administration of propionate although the extent of this infiltration was dependent on the time of administration (Fig. 8A and Supplementary data, Fig. S9A). No changes in T cell and macrophage subsets were detected upon renal injury or after propionate treatment (Supplementary data, Fig. S9B and C). Reduced infiltration of neutrophil and monocyte cells within the kidney was associated with decreased expression of chemokines such as *Ccl2, Ccl5, Cxcl2* and *Cxcl3*, and cytokines *Il6* and *Tnf* (Fig. 8B). The effect upon these pro-inflammatory molecules was dependent on when propionate was administered.

**Figure 9:**
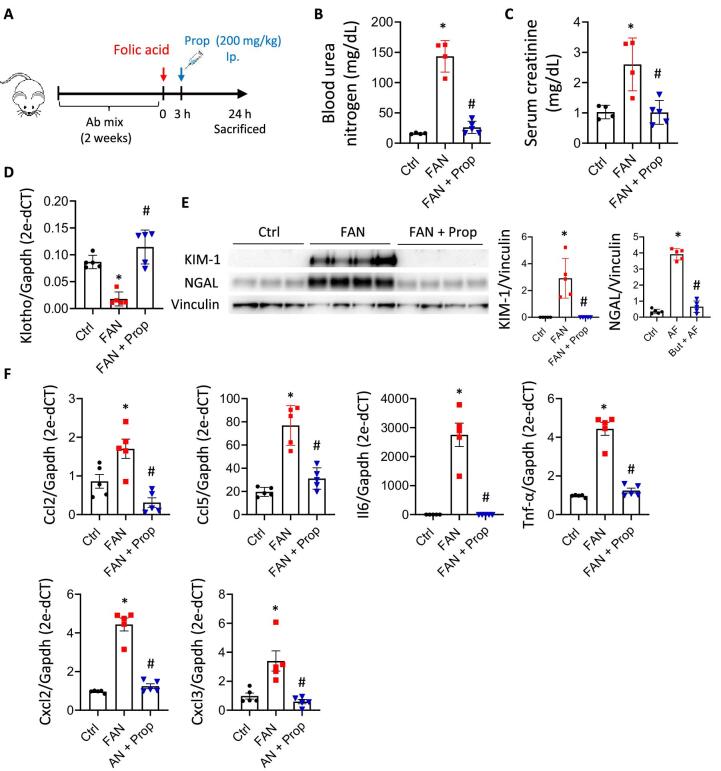
Propionate (Prop) and butyrate (But) alleviate renal damage independently of the gut microbiota. (**A**) Graphic representation of the renal damage model induced in mice pre-treated with broad-spectrum antibiotics (Ab mix) for 2 weeks, followed by ip administration of a high dose of folic acid (250 mg/kg) and Prop (200 mg/kg, 3 h after FA). Mice were sacrificed 24 h after damage. Groups: control (Ctrl; *n* = 3–5), folic acid nephropathy (FAN; *n* = 4–5), FAN treated with Prop (FAN + Prop; *n* = 5). Quantification of the serum levels of BUN (**B**) and creatinine (**C**). (**D**) Quantification of the transcriptional levels of klotho. (**E**) Determination of protein levels of the renal injury markers KIM-1 and NGAL in kidney tissue by western blotting. (**F**) Expression of pro-inflammatory molecules in kidney tissue analysed by RT-PCR in the different groups. *Gapdh* and vinculin were used as endogenous controls for RT-PCR and western blotting, respectively. Data are shown as the mean ± SD. *P < .05 vs Ctrl; ^#^*P* < .05 vs FAN as determined by the Mann–Whitney test.

To investigate whether the effect of the propionate treatment is independent of the intestinal flora, we used the same approach of administering propionate 3 h after renal damage induction, but in mice previously treated with broad-spectrum antibiotics to deplete their original intestinal flora (Fig. 9A). Again, we observed that early administration of propionate enables recovery of renal function (creatinine and BUN levels), klotho expression, and reduction of the KIM-1 and NGAL renal damage markers while maintaining a low level of expression of pro-inflammatory molecules (Fig. 9B–F). To corroborate these findings, propionate was additionally administered to mice with an intact microbiota at the same doses but by oral gavage. The results were compared with those of the mouse group with ip administration of propionate (Supplementary data, Fig. S10A). It is of particular note that no effect was observed on renal function, klotho expression or damage markers when propionate was orally administered (Supplementary data, Fig. S10B–F). Therefore, we can conclude that these metabolites have a direct effect in the kidney after ip administration that is independent of the gut microbiota, as evidenced by the similar levels of bacterial groups and propionate levels in cecum samples (Supplementary data, Fig. S10G and H). Only a few specific changes were observed in Lactobacillus and Enterobacteriaceae, but these were probably related to the renal damage rather than to the administration of propionate.

## DISCUSSION

There is strong evidence to show that the gut microbiota influences renal pathology. However, despite all these studies, the direct impact of the SCFAs, independent of the gut microbiota, has not been fully clarified. Here, we have shown that propionic and butyric acid levels are gradually reduced in CKD patients as the disease progresses and are closely correlated with clinical parameters of renal function, supporting the bidirectional relationship between the gut and kidney. Additionally, we have demonstrated that early administration of propionate and butyrate after injury, and within a defined therapeutic window, limits recruitment of myeloid cells to the kidney and reduces the inflammatory and pro-fibrotic responses. Therefore, these SCFAs can directly modulate the kidney, without inducing changes in the gut microbiota, and can regulate a variety of maladaptive processes triggered immediately after AKI that contribute to subsequent chronic renal damage.

The many studies carried out so far have identified quantitative and qualitative alterations in the gut microbiota of CKD patients that are associated with the production of their microbial metabolites [39–41]. The balance between competing bacterial groups had been confirmed by the negative correlation between faecal levels of butyric acid and the serum levels of some uraemic toxins, such as p-cresyl sulfate, p-cresyl glucuronide and trimethylamine N-oxide [42, 43]. In our study, no changes in the bacterial composition were detected in CKD patients and only a slight reduction of *Lactobacillus* was reported, providing further evidence of its beneficial role [44]. Disparities between studies could be due to the different composition of the populations analysed, and we cannot rule out the possibility that further studies based on microbiome sequencing could provide us with more details about the microbial diversity in these patients. However, we detected a clearly significant and concomitant reduction of the propionate and butyrate levels as the disease progressed. Previously, Wang *et al*. [43] reported a similar result in a larger cohort of CKD patients, but they only detected low levels of butyrate in advanced (CKD5) stages of the disease compared with early (CKD1–4) stages and healthy controls. Thus, to the best of our knowledge, this is the first time that the propionate levels have also been associated with the progressive loss of renal function. Wang *et al*. [43] also reported a reduced faecal concentration of butyrate-high-producing bacteria, such as the phylum Firmicutes, but no association with butyrate levels was described. Thus, we speculate that the determination of SCFAs levels in these patients could be a more homogeneous and reliable marker than the quantification of bacteria due to the strong influence of diet and food habits on microbiota diversity and to cross-feeding. In the latter phenomenon, some bacteria are fed with SCFAs produced by other bacteria, which makes it more difficult to demonstrate a direct correlation between SCFA levels and their bacteria of origin [45].

It is clearly established that low-grade systemic inflammation contributes to CKD development. In our study, we observed elevated serum levels of TNF-α and CXCL8 (IL-8), but no other assayed cytokines, in patients with advanced CKD, as previously reported [46, 47]. However, this was not correlated with SCFA levels. Inflammation in CKD is a complex condition that is influenced by a variety of patient-related factors, such as the underlying diseases, comorbidities, infections, and the genetic and lifestyle factors connected to diet and microbiota [48]. Thus, taking all these variables into account could help determine the contribution of inflammation to renal function decline before reaching end-stage renal disease, whereupon a more homogeneous condition is probably established because of the significant contribution to the disease.

Here, a well-established model of AKI-to-CKD transition induced by folic acid was used to evaluate the restoration of the SCFA levels in the progression of renal disease. In this process, multiple lines of evidence have shown that TECs drive the inflammatory and pro-fibrotic phenotypes that aggravate the damage [49]. By analysing the transcriptional profile of these cells under inflammatory conditions, we found that the changes induced by propionate and butyrate point in two main directions: downmodulation of pro-inflammatory molecules and recruitment of immune cells. These changes were similar when metabolites were added before or after TNF-α activation, suggesting for the first time that these compounds are able to revert, rather than just prevent, inflammation. These results demonstrate that propionate and butyrate can prevent the immune and inflammatory responses triggered as a consequence of an initial injury, avoiding the development of fibrosis over the long term. Even more significantly, these metabolites are able to curb the loss of renal function once it has been initiated.

Propionate was found to impair recruitment of neutrophils and monocytes and was dependent upon administration time. It was also correlated with decreased expression in the kidney of chemokine genes responsible for attracting these myeloid cells, such as *Cxcl2, Cxcl3, Ccl2* and *Ccl5*. Recruitment of neutrophils is an early event in AKI that exacerbates further infiltration of monocytes/macrophages and aggravates and perpetuates chronic inflammation in CKD progression [50]. Butyrate not only reduces the migration of neutrophils to the sites of damage in different pathological contexts, but also interferes with neutrophil extracellular trap formation and response to oxidative stress [51, 52]. Thus, reducing the infiltration of these myeloid cells in the first hours after injury could be essential to impeding exacerbated inflammation. We hypothesized that the dampening of the inflammatory response is indeed responsible for the long-term recovery of the kidney, as has been previously reported [53]. However, we cannot discount the possibility that these metabolites initially protect the epithelial cells from damage, with a consequent reduction in the inflammatory response. To rule out this alternative explanation, experiments using single-cell techniques to determine the responses of various cell types to SCFAs, in a time-dependent context, should be conducted.

Previously, pre-clinical studies had shown that maintaining sufficient SCFA levels, either by feeding with high-fibre diets or through direct supplementation into drinking water, can help preserve the function of kidneys damaged by ischemic acute injury or glomerular pathology [24, 25, 54]. More recently, supplementation with the probiotic *Lactobacillus casei* Zhang in murine models and individuals with CKD showed an improvement in kidney function due to the anti-inflammatory properties of SCFAs and nicotinamide [27]. All these investigations have primarily focused on the effects mediated by the SCFAs because of changes induced in gut microbiota composition. In contrast, our investigation took a novel approach by evaluating the direct impact of these microbial metabolites (propionate and butyrate) on the mechanisms triggered in the kidney following damage.

Using the same model of renal damage, Liu *et al*. [26] showed that the continuous administration of SCFAs in drinking water, beginning 2 weeks before damage and maintained throughout the study, led to a partial recovery of renal function over the long term (28 days). Our study provides valuable new insight by describing that the administration of propionate or butyrate only on the days immediately before and after peak acute injury (from –24 h until +72 h) allows long-term preservation of renal function. Therefore, these findings considerably narrow the optimal intervention window for SCFAs, indicating that the days around an acute injury are crucial if maximum benefit is to be gained. Similar results were observed when both microbial metabolites were administered once damage had been initiated: the observed rapid recovery of renal function helps establish a therapeutic window for the use of these metabolites as treatment. This delineation of the timing of SCFA administration for maximum efficacy represents a major advance in therapeutic strategy.

The initial phase of folic acid–induced damage is characterized by a maladaptive repair leading to atrophic proximal tubules and renal interstitial fibrosis [55]. Multiple mechanisms triggered in the TECs, such as oxidative stress, cell death, cell cycle arrest and defective tubular regeneration, are involved. Andrade-Oliveira *et al*. [24] showed that acetate reduces apoptosis in tubular cells, inhibits production of reactive oxygen species and activates autophagy. Results from the mRNA-sequencing studies allowed us to identify a large number of genes involved in these pathways that are modulated by propionate and butyrate. However, the exact role of these SCFAs in each pathway will require further investigations to reveal hitherto unidentified routes of action of these metabolites that are independent of the inflammation.

The two microbial metabolites had an identical impact, suggesting that their mechanism of action might be mediated by epigenetic changes via HDAC inhibition or activation of acetyltransferase p300 [56] since these properties are shared by the two metabolites. This was confirmed by the changes observed in genes involved in the chromatin remodelling, and specifically in histone acetylation, identified from the sequencing analysis of TECs. One example is the recovery of klotho levels, a renoprotective protein the expression of which is highly dependent on its acetylation level. Mice treated with propionate at different times after damage showed partially lowered downregulation of klotho. Similarly, it has been reported that inhibition of HDAC3 and HDAC8 derepressed klotho expression during renal fibrosis [57, 58]. However, effects mediated by activation of GPRs, such as GPR43 expressed by distal renal tubules and collecting tubules, cannot be ruled out. In fact, Liu *et al* [26], using GPR knockout mice, showed that the effect on renal function mediated by a high-fibre diet or butyrate was lost when GPR109 and GPR41 were absent. The depletion of these receptors was not kidney specific, thus it should be explored to obtain a more accurate knowledge. Furthermore, crosstalk between GPR signalling activation and HDAC inhibition might also occur, which is indicative of the great complexity of the regulatory mechanisms mediated by SCFAs.

We acknowledge that this study has some limitations. Although our model of FAN has been extensively used to study AKI-to-CKD transition [59], it does perfectly mimic human CKD. Additionally, we chose the ip administration route with the aim of reaching the kidney more efficiently and demonstrating its direct impact, independent of the influence of gut microbiota. It is known that these metabolites, when administered orally, are rapidly degraded in the gastrointestinal tract and have extremely short half-lives. Once we know the potential of these metabolites to alleviate renal damage, but before translating these results to clinical practice, novel delivery systems should be developed, such as the use of SCFA-containing nanoparticles that are targeted to tubular cells [60, 61]. On the other hand, we cannot accurately estimate the dose required for human oral supplementation. Using a Food and Drug Administration–recommended method for dose conversion between mice and humans [62], we estimated that the doses used in this study were similar to those proposed in several ongoing clinical trials cells [63]. However, further adjustments regarding the bioavailability, absorption rates and metabolic differences between administration routes still need to be considered. One important question arising from our work is whether increasing SCFAs levels in the intermediate stages of CKD would help slow down progression of the disease. This is particularly pertinent as we only treated the mice shortly after damage. This intriguing situation emphasizes the need for further research to elucidate the complex mechanisms regulated by SCFAs during the development of CKD.

In summary, the close relationship between butyrate and propionate levels and renal function in CKD patients shows the importance of these metabolites in renal damage. Using a pre-clinical model of AKI-to-CKD transition, we demonstrate that the early administration of these SCFAs after injury allows renal function to be restored and progression of the disease to be avoided. These results suggest new avenues for exploring the potential therapeutic value to renal pathology of propionate and butyrate, independent of the gut microbiota.

## Supplementary Material

gfae118_Supplemental_Files

## Data Availability

The data on which this article is based are available in the article and in its online supplementary material. Sequencing data has been deposited on the Gene Expression Omnibus repository (GEO: GSE221506). To review the sequencing data, go to https://www.ncbi.nlm.nih.gov/geo/query/acc.cgi?acc=GSE221506.
