# Supplementary material for: Propionate and butyrate counteract renal damage and progression to chronic kidney disease
Source: Nephrol Dial Transplant. 2024 May 24;40(1):133–50. doi: 10.1093/ndt/gfae118 (PMC11852269; doi:10.1093/ndt/gfae118)
Supplement: gfae118_Supplemental_Files [file gfae118_Supplemental_Files.zip › Supplementary Figures.pdf]

Supplementary Figure 1

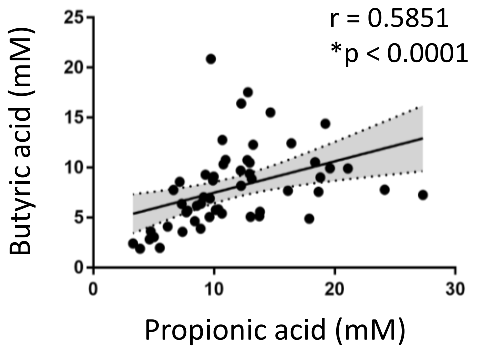

Supplementary Figure 1.- Propionic and butyric acids show similar profiles during chronic kidney disease (CKD) progression. Correlation between propionic and butyric acid levels obtained from faecal samples of CKD patients (n = 54). Spearman correlation coefficients were calculated; values of $p < 0.05$ were considered statistically significant (*).

Supplementary Figure 2

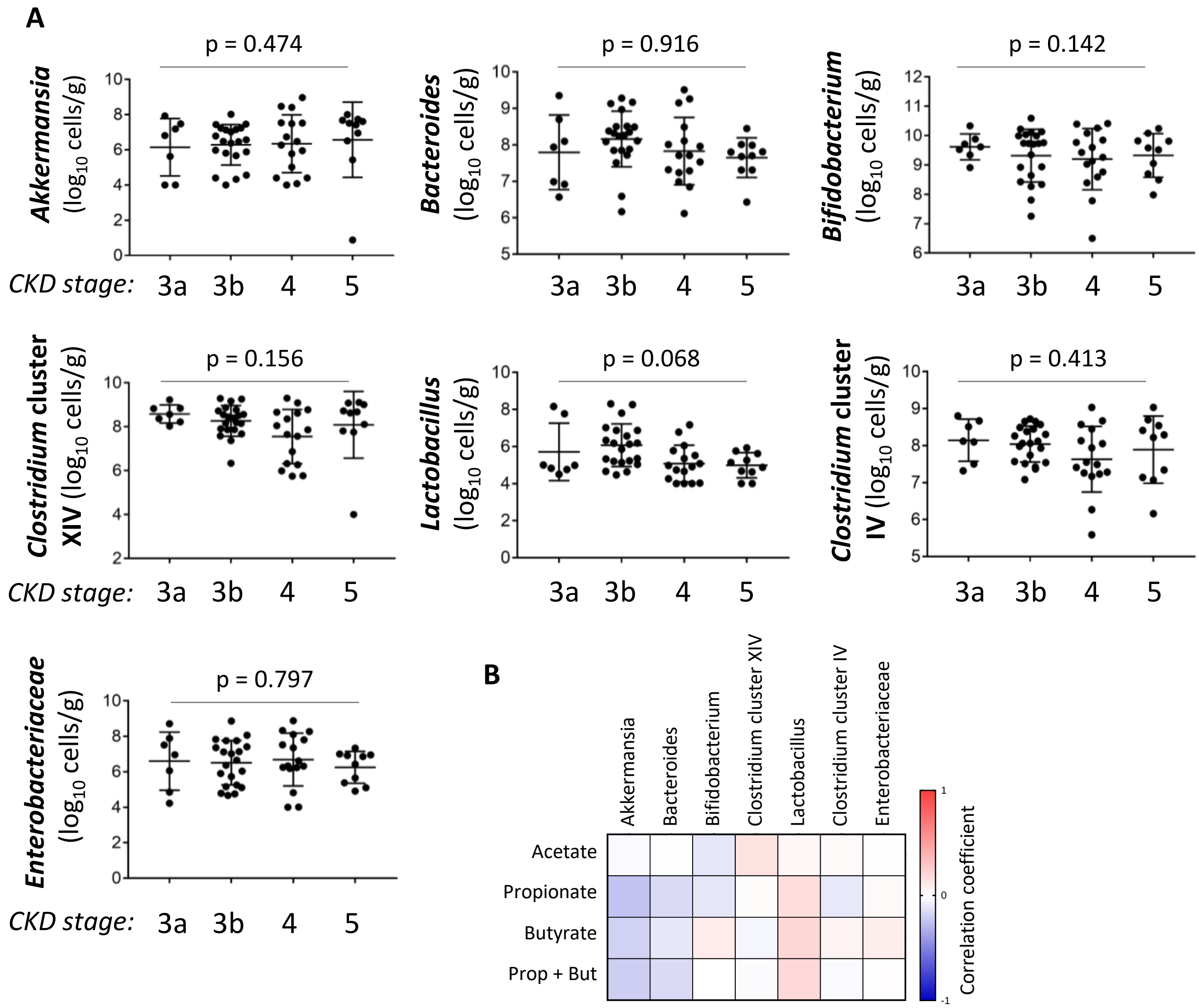

Supplementary Figure 2.- Gut microbiota profiles in chronic kidney disease (CKD) patients at different stages of disease. (**A**) Bacterial levels of diverse intestinal microbial groups (*Akkermansia*, *Bacteroides* group, *Bifidobacterium*, *Clostridium cluster XIVa*, *Lactobacillus* group, *Clostridium cluster IV*, and Enterobacteriaceae) in faecal samples from patients at different CKD KDIGO stages; 3a (n=7), 3b (n=21), 4 (n=16), and 5 (n=10). Data were calculated as the log of the number of cells per gram of faeces. Values are shown individually and the mean ± standard deviation. Groups were compared using a Kruskal-Wallis test; values of $p < 0.05$ indicate significant differences (*). (**B**) Heatmap showing the correlation between acetic, propionic, butyric, and propionic + butyric acids and intestinal microbial groups. Positive and negative Spearman correlations are indicated in red and blue, respectively. The colour intensity is proportional to the strength of the association.

Supplementary Figure 3

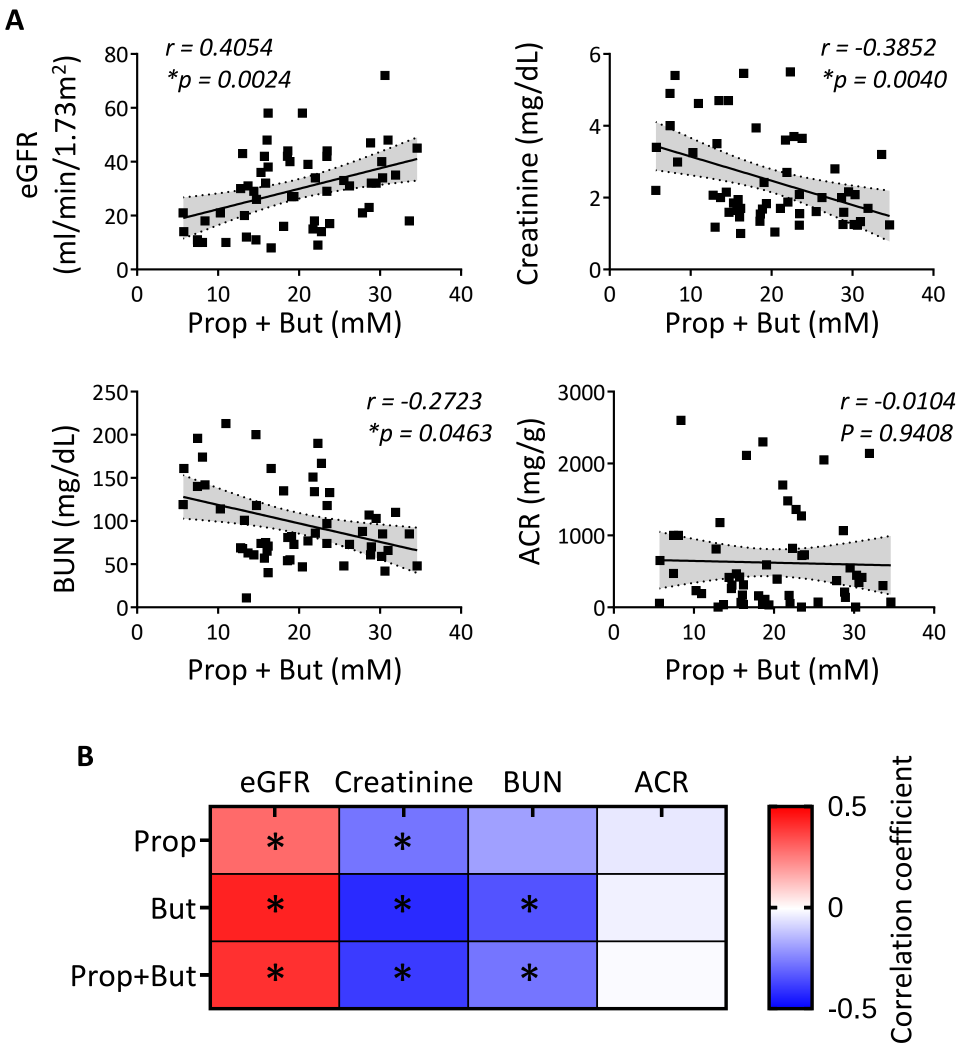

Supplementary Figure 3.- Correlation between the propionic plus butyric acid levels and renal clinical parameters in chronic kidney disease (CKD) patients. (A) Correlation between the absolute concentration (mM) of propionic + butyric acid and the levels of estimated glomerular filtration rate (eGFR), creatinine, blood urea nitrogen (BUN) and albumin/creatinine ratio (ACR) levels in CKD patients (n=54). (**B**) Heatmap of Spearman correlation coefficients between metabolites (propionic and/or butyric acids) and clinical parameters in CKD patients. Values of $p < 0.05$ (*) indicate statistically significant coefficients.

Supplementary Figure 4

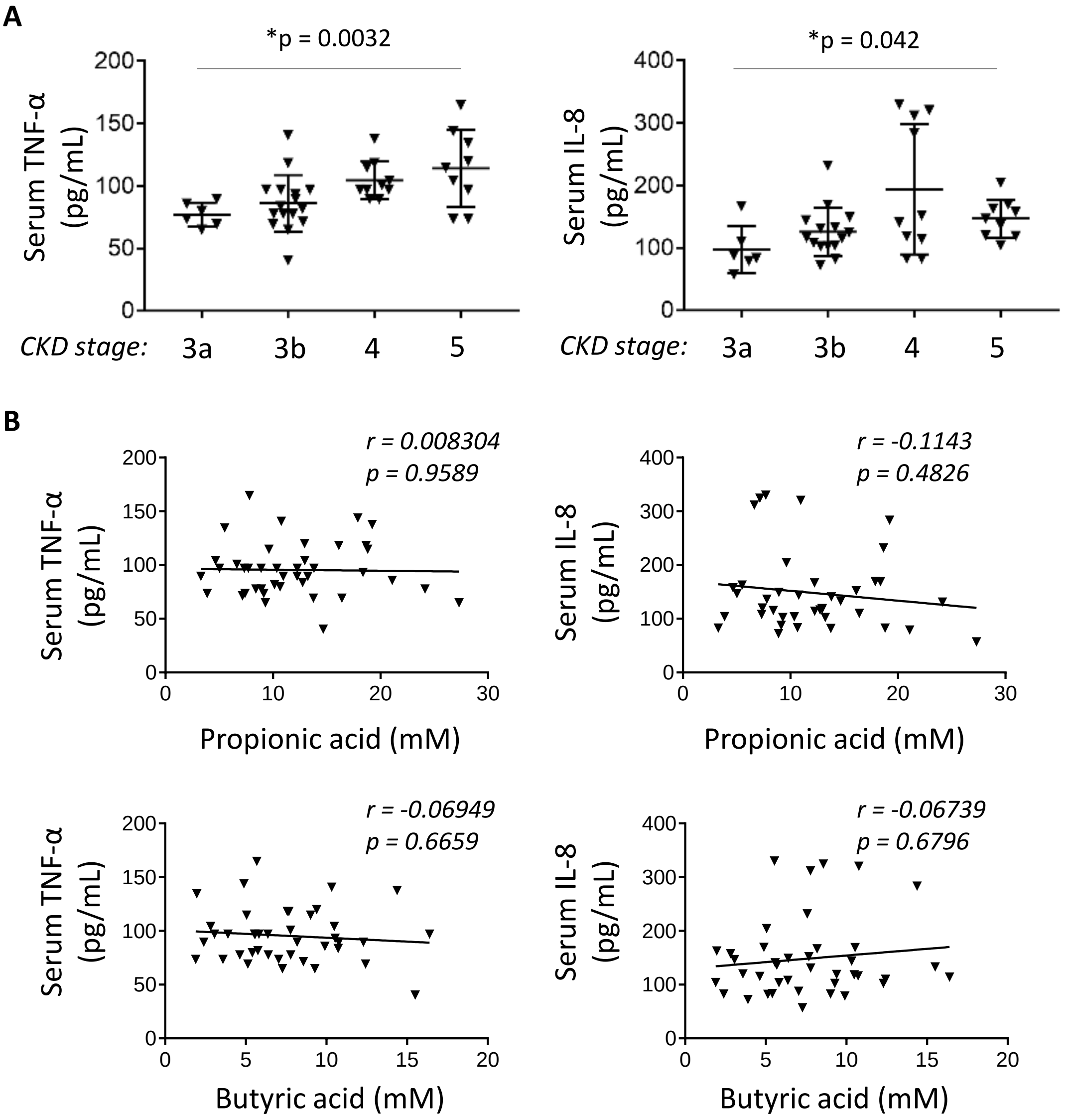

Supplementary Figure 4.- Levels of TNF-α and IL-8 pro-inflammatory cytokines in chronic kidney disease (CKD) patients and correlation with short-chain fatty acids. (**A**) TNF-α and IL -8 cytokines were assayed in serum of CKD patients at different stages of their disease: 3a (n=7), 3b (n=21), 4 (n=16), and 5 (n=10). Values are shown individually and the mean ± standard deviation. Groups were compared using Kruskall-Wallis test, values of $p < 0.05$ (*) indicate tatistically significant differences. (**B**) Correlation between propionic or butyric acids levels obtained from faecal samples and serum levels of TNF-α and IL-8 cytokines in CKD patients (n=54). Spearman correlation coefficients were calculated. Values of $p < 0.05$ (*) indicate statistically significant coefficients.

Supplementary Figure 5

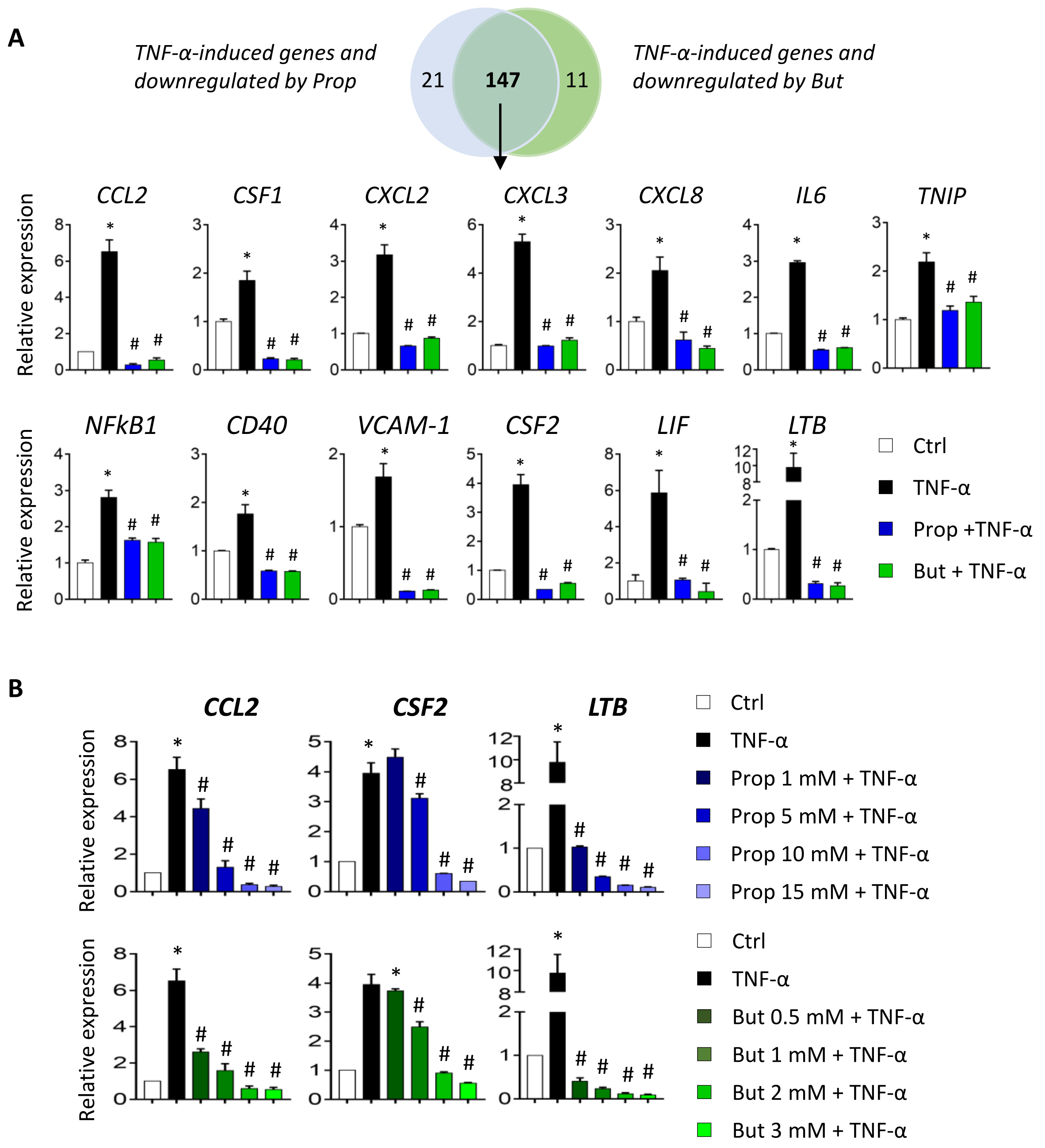

Supplementary Figure 5.- Propionate and butyrate downregulate the inflammatory profile induced by TNF-α in a dose-dependent manner. (**A**) Venn diagrams showing the common genes (n=147) induced by TNF-α and downregulated by propionate and butyrate. Histograms show the gene expression of representative genes quantified by RT-PCR. (**B**) Expression of *CCL2*, *CSF2* and *LTB* was assayed by RT-PCR in the HK2 cell line after treatment for 24 h with different doses of propionate (Prop; 1, 5, 10, and 15 mM) and butyrate (But; 0.5, 1, 2, and 3 mM) following induction of inflammation with TNF-α treatment (3 h; 5 ng/ml). *Gapdh* was used as an endogenous control. Data are shown as the mean ± standard deviation of two independent experiments and were compared using the t-Studnet test. *p < 0.05 *vs.* control; #p < 0.05 vs TNF-α-treated cells.

Supplementary Figure 6

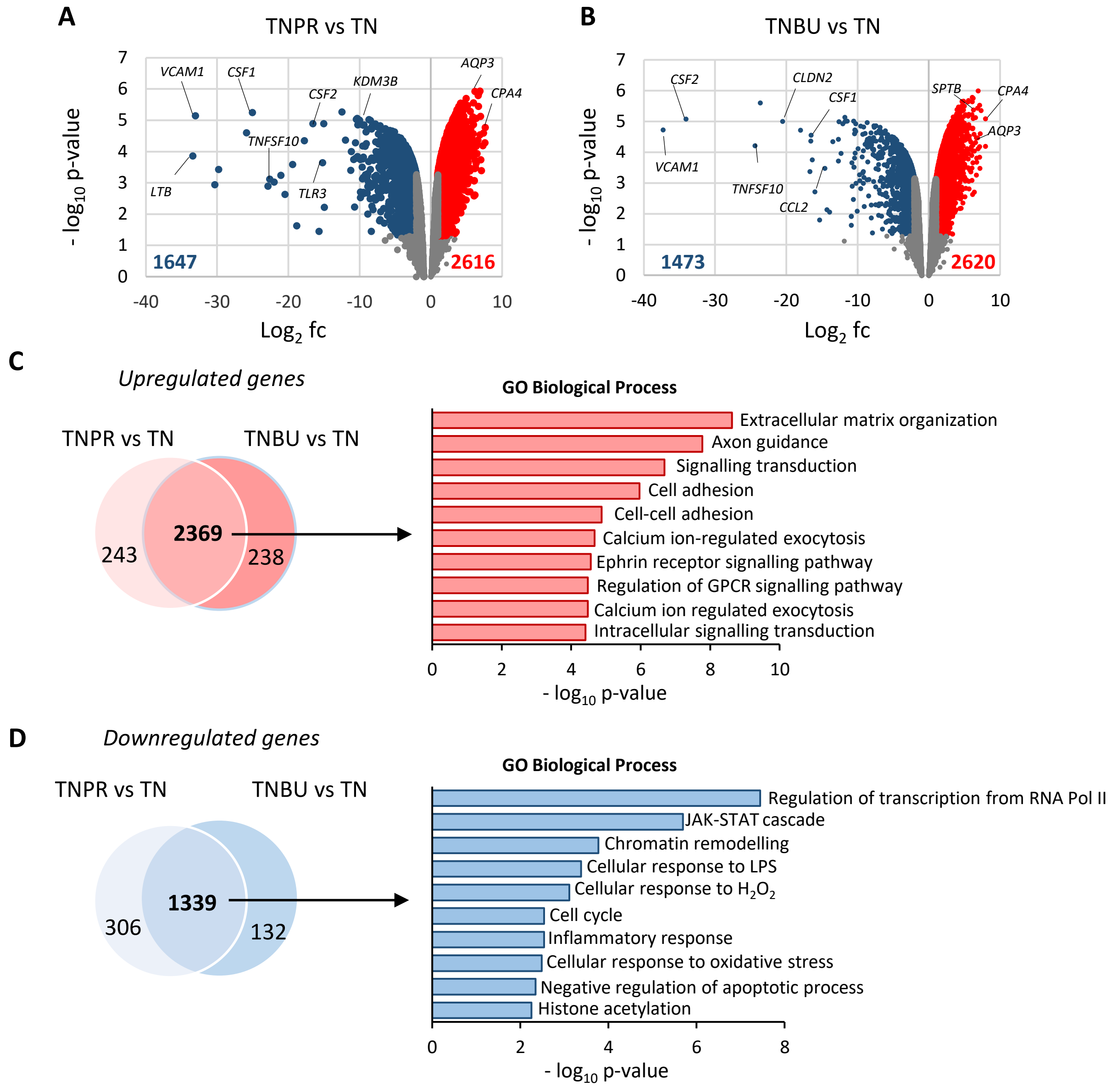

Supplementary Figure 6.- Propionate and butyrate induce transcriptional changes in tubular epithelial cells independent of TNF-α. Volcano plots of the TNPR vs TNF (**A**) and TNPR vs TNF (**B**) comparisons showing the differentially expressed genes (DEGs) with both SCFA independently of TNF-α induction. Downregulated and upregulated genes fulfilling the crietria of a > 2-fold change (fc) and adjusted $p < 0.05$ are shown in blue and red, respectively. Some of the most significant DEG are illustrated. Venn diagrams showing the upregulated (**C**) and downregulated (**D**) genes for the different condictions, enabling the identification of a commom signature of genes induced or repressed by both SCFA independently of TNF-α activation. Biological processes associated with each signature were identified. TN, TNF-α; PR, propionate; BU, butyrate.

Supplemental Figure 7

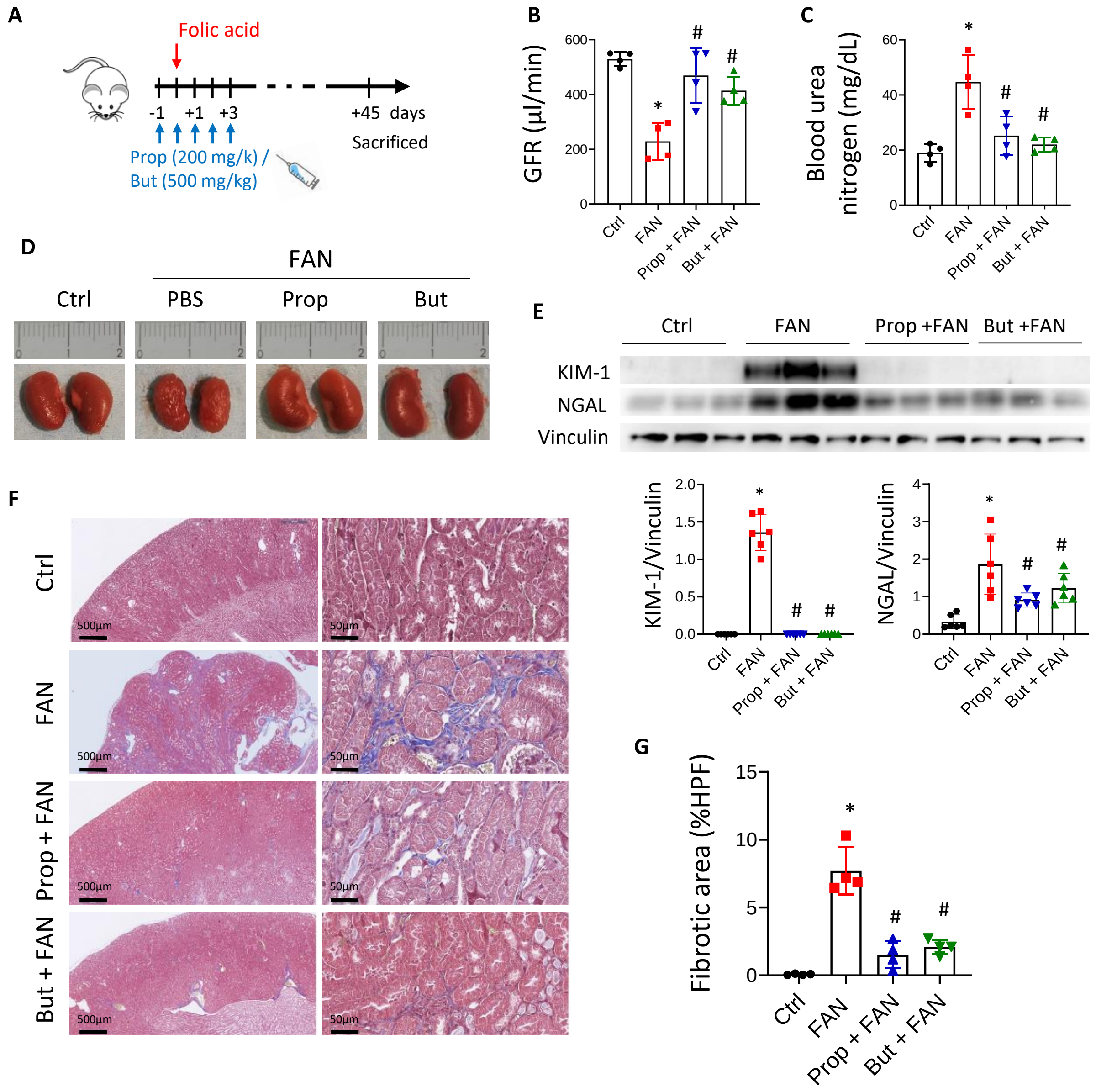

Supplementary Figure 7.- Pre-treatment with propionate (Prop) and butyrate (But) prevents the development of fibrosis at long term. (**A**) Graphic representation of a mouse model of renal damage induced by high doses of folic acid (250 mg/kg). Prop (200 mg/kg) and But (500 mg/kg) were independently administered before damage, beginning one day before (day -1) and every 24 h for five consecutive doses until day +3. Renal function and kidney tissue were analysed at day +45 in the following groups: control (Ctrl; n= 6), folic acid nephropathy (FAN; n=6), FAN pre-treated with Prop (Prop + FAN; n=8), and FAN pre-treated with But (But + FAN; n=8). Quantification of the transcutaneous glomerular filtration rate (GFR; µl/min) (**B**) and blood urea nitrogen (mg/dL) (**C**) at day +45; n=3-4 mice per group. (**D**) Representative images of kidneys from different groups. (**E**) Protein levels of the renal damage markers KIM-1 and NGAL analysed by western blotting; n=6 mice per group. Vinculin was used as an endogenous control. Data are shown individually and the mean ± standard deviation. *$p < 0.05$ *vs.* control; #p < 0.05 *vs.* FAN as determined by the Mann-Whitney test. (**F**) Representative images of Masson's trichrome staining in the different indicated groups and their corresponding quantification of the fibrotic area (%). Scale bar is indicated in the images. Data are shown individually and the mean ± standard deviation. *$p < 0.05$ *vs.* control; #p < 0.05 *vs.* FAN as determined by the Mann-Whitney test.

Supplementary Figure 8

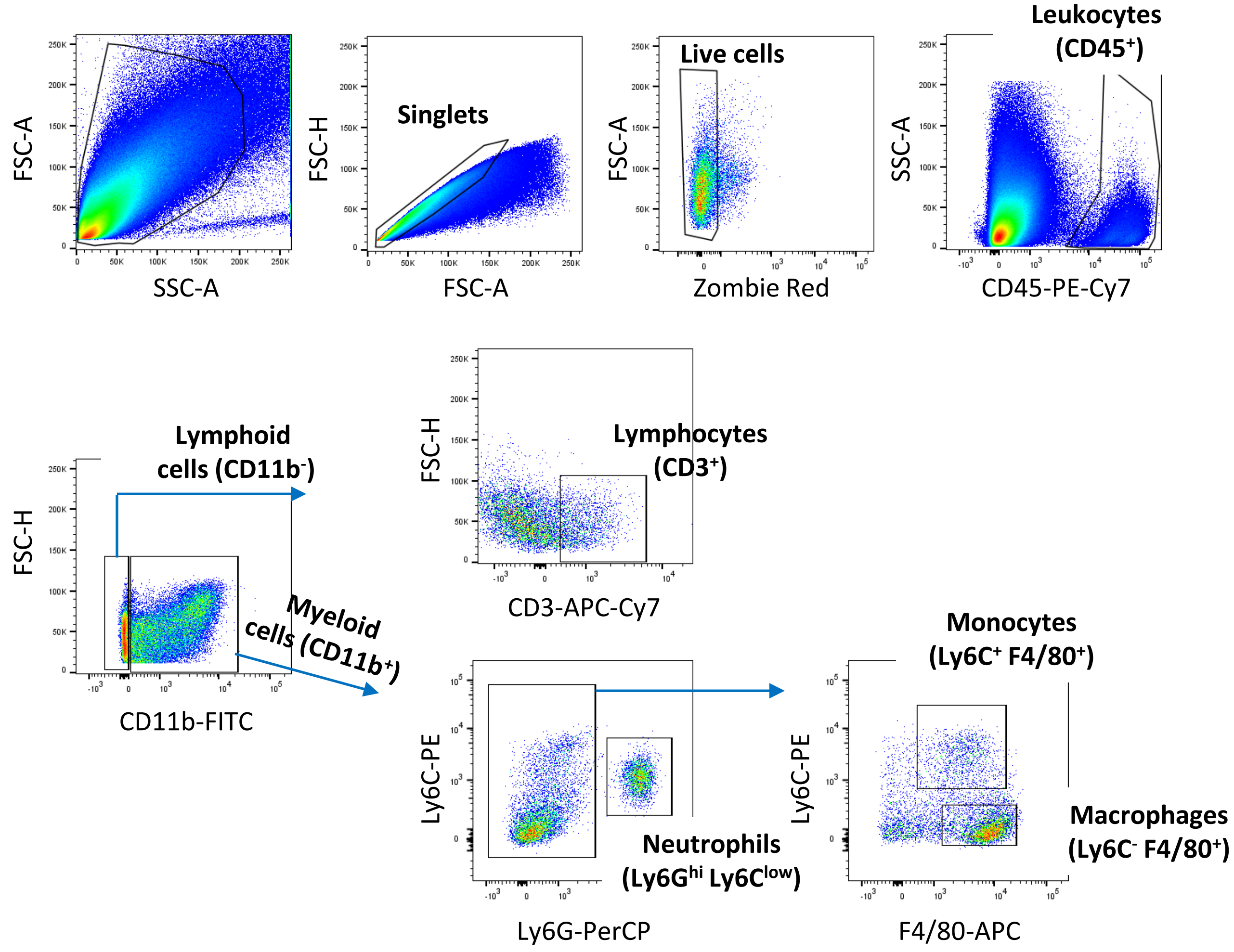

Supplementary Figure 8.- Flow cytometry gating strategy in kidney tissue. Gatting strategy for analysing lymphoid and myeloid cell subsets infiltrating kidney tissue. Initially, debris, doublets, and dead cells (Zombie Red-negative cells) were excluded from the minced kidneys. The leukocyte population was selected using the CD45 marker. Lymphoid ($CD11b^-$) and myeloid ($CD11b^+$) cells were identified with the CD11b marker. Lymphocytes ($CD3^+$) were selected from lymphoid cells using the CD3 marker. From the myeloid cells ($CD45^+$ $CD11b^+$), neutrophils were identified according to high Ly6G and low Ly6C expression. Using the F4/80 marker, monocytes ($Ly6C^+$ $F4/80^+$) and macrophages ($Ly6C^-$ $F4/80^+$) were distinguished within the non-neutrophil population.

Supplementary Figure 9

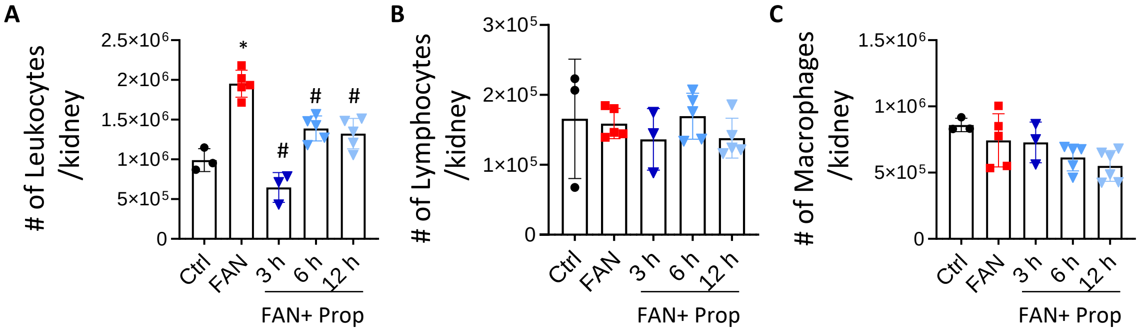

Supplementary Figure 9.- Infiltration of immune cells after propionate (Prop) treatment. A renal damage was induced in a mouse model with high doses of folic acid (250 mg/kg). Prop (200 mg/kg) was intraperitoneally administered at 3 h, 6 h, and 12 h after folic acid. Cells infiltrating the kidney were analysed 24 h after initial damage. Groups were control (Ctrl; n=3), folic acid nephropathy (FAN; n=5), FAN + Prop 3h (n=4), FAN + Prop 6h (n=5), and FAN + Prop 12h (n=5). Leukocytes, lymphocytes, and macrophages infiltrating the kidney were quantified by flow cytometry. Results are shown as the total cell number per kidney. The complete gating strategy is shown in **Fig. S8.** Data are represented individually and the mean ± standard deviation. *$p < 0.05$ *vs.* control; #$p < 0.05$ *vs.* FAN as determined by the Mann-Whitney test.

Supplementary Figure 10

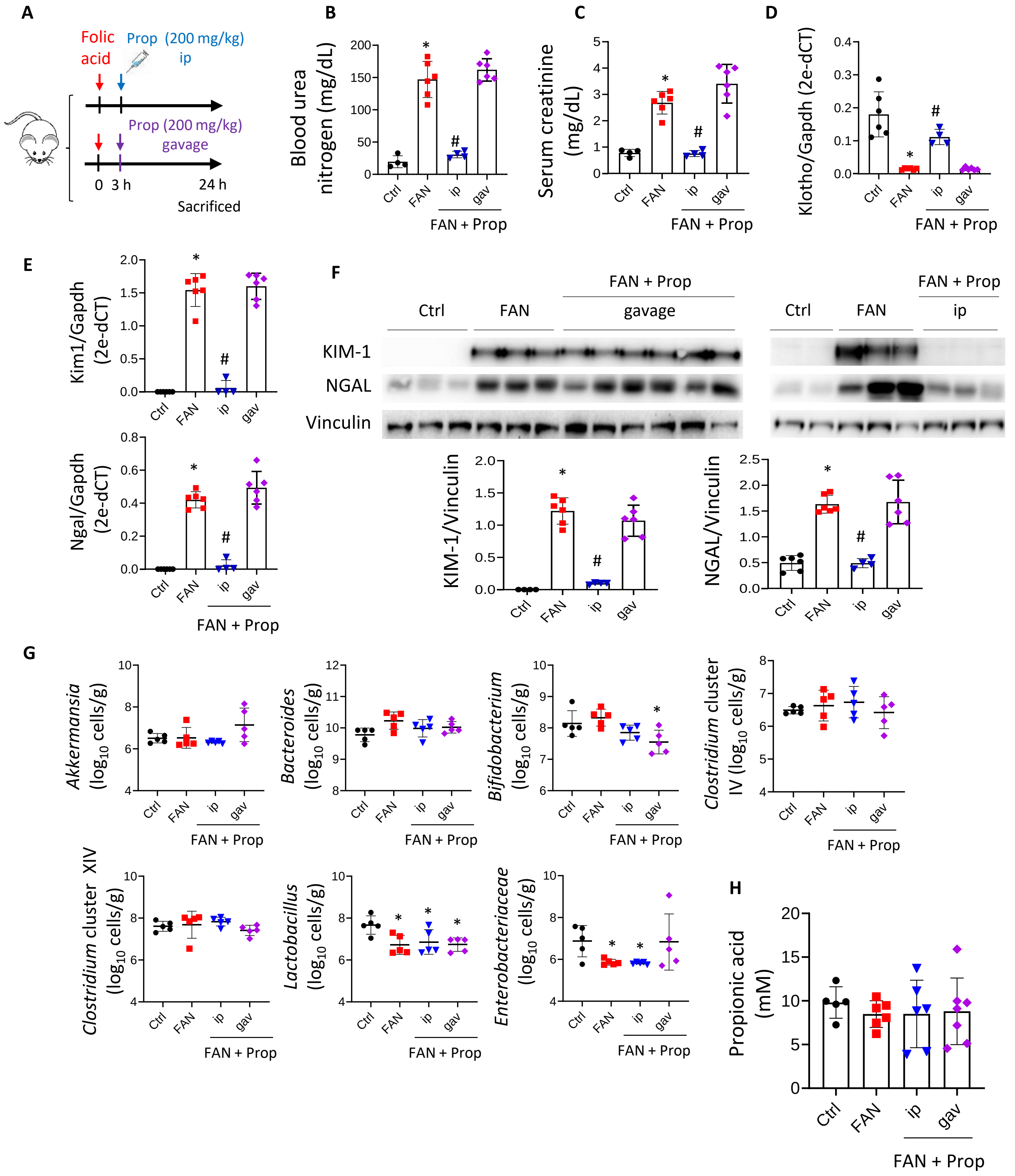

Supplementary Figure 10.- The administration route is key to mediating the effect of Propionate (Prop) on the renal function. (**A**) Graphic representation of the renal damage model induced by high doses of folic acid (250 mg/kg) followed by Prop (200 mg/kg) administration 3 h after damage, by intraperitoneal (ip) injection or gavage (gav). Mice were sacrificed at 24 h after damage. Groups: control (Ctrl; n= 4-6), folic acid nephropathy (FAN; n=6), FAN treated with Prop via ip (FAN + Prop ip; n=4-6), and FAN treated with Prop via gavage (FAN + Prop gav; n=6). Quantification of the serum levels of blood urea nitrogen (**B**) and creatinine (**C**). (**D**) Quantification of the transcriptional levels of klotho. (**E,F**) Determination of transcriptional and protein levels of the renal injury markers KIM-1 and NGAL in kidney tissue by western blotting. *Gapdh* and vinculin were used as endogenous controls for RT-PCR and western blotting, respectively. (**G**) Bacterial levels of the different microbial groups in cecum samples from mice of the different groups. Data were calculated as the log of the number of cells per gram of faeces. (**H**) Levels of propionate (mM) in cecum samples from the different groups. Data are shown individually and as the mean ± standard deviation. *$p < 0.05$ *vs.* Ctrl; #$p < 0.05$ *vs.* FAN as determined by the Mann-Whitney test.
