# Supplementary material for: Propionate and butyrate counteract renal damage and progression to chronic kidney disease
Source: Nephrol Dial Transplant. 2024 May 24;40(1):133–50. doi: 10.1093/ndt/gfae118 (PMC11852269; doi:10.1093/ndt/gfae118)
Supplement: gfae118_Supplemental_Files [file gfae118_Supplemental_Files.zip › Table S1.pdf]

Supplemental Table 1.- Primers sequences and Taqman assays

Name	Chemistry	Species	Dye	Assay identification
CCL2	Taqman	Human	FAM-MGB	Hs00234140_m1
CXCL8	Taqman	Human	FAM-MGB	Hs01038788_m1
GAPDH	Taqman	Human	VIC-MGB	Hs02758991_g1
IL6	Taqman	Human	FAM-MGB	Hs00174131_m1
LTB	Taqman	Human	FAM-MGB	Hs00242739_m1
Gapdh	Taqman	Mouse	VIC-MGB	Mm99999915_g1
Kim1	Taqman	Mouse	FAM-MGB	Mm00506686_m1
Il6	Taqman	Mouse	FAM-MGB	Mm00446190_m1
Ngal	Taqman	Mouse	FAM-MGB	Mm01324470_m1

Name	Chemistry	Species	Forward sequence (5' to 3')	Reverse sequence (5' to 3')
CD40	SYBR Green	Human	CACCAGCACAAATACTGCGAC	GTGTCTGTTTCTGAGGTGGC
CSF1	SYBR Green	Human	CTCTCCGCATCCCAGGACAG	AGACCAACAACAGCAGGGAG
CSF2	SYBR Green	Human	TCCTGAACCTGAGTAGAGACAC	TGCTGCTTGTAGTGGCTGG
CXCL2	SYBR Green	Human	GCTTGTCTCAACCCCGCATC	TCTGGTCAGTTGGATTTGCCATTTT
CXCL3	SYBR Green	Human	CATCCAAAGTGTGAATGTAAGGT	CCATTCTTGAGTGTGGCTATGAC
GADPH	SYBR Green	Human	TGCCATGGGTGGAATCATATTGGA	TCGGAGTCAACGGATTTGGTCGT
LIF	SYBR Green	Human	CCACCCATGTCACAACAACC	CTATTACACAGCCCAGGGGG
NFKB1	SYBR Green	Human	AGCAGATGGCCCATACCTTC	CAGGTAGTCCACCATGGGATG
TNIP	SYBR Green	Human	CCTGAGGAGCAGAATTCACCA	GCATCAGGTTGCCGTCCT
VCAM1	SYBR Green	Human	GGGAAGATGGTCGTGATCCTT	TCTGGGGTGGTCTCGATTTTA
Acta2/a-Sma	SYBR Green	Mouse	ACTGGGACGACATGGAAAAG	GTTCAGTGGTGCCTCTGTCA
Ccl2	SYBR Green	Mouse	GATGCAGTTAACGCCCCACT	CCCATTCCTTCTTGGGGTCA
Ccl5	SYBR Green	Mouse	TGCTTTGCCTACCTCTCCCT	ACACACTTGGCGGTTCCTTC
Cd40	SYBR Green	Mouse	GAGGTCCTACAGAAAGGAACGA	TGCCCATCACGACAGGAATG
Col1a1	SYBR Green	Mouse	ACATGTTCAGCTTTGTGGACC	TAGGCCATTGTGTATGCAGC
Csf1	SYBR Green	Mouse	AGACTTCATGCCAGATTGCCT	AAGCGCATGGTCTCATCTATT
Cxcl2	SYBR Green	Mouse	CCAACCACCAGGCTACAGG	GCGTCACACTCAAGCTCTG
Cxcl3	SYBR Green	Mouse	CTGCACCCAGACAGAAGTCAT	ATTATCTGAAGCCTGGGGCCT
Fib	SYBR Green	Mouse	GGAGTGGCACTGTCAACCTC	ACTGGATGGGGTGGGAAT
Fsp1	SYBR Green	Mouse	CAGCACTTCCTCTCTCTTGG	TTTGTGGAAGGTGGACACAA
Gapdh	SYBR Green	Mouse	TGTGTCCGTCGTGGATCTGA	TTGCTGTTGAAGTCGCAGGAG
Klotho	SYBR Green	Mouse	GCC CAC AAC CTA CTT TTG GC	GAT AGA CAC CCG GCC TCC C
Ltb	SYBR Green	Mouse	AGGGACGTCGGGTTGAGAA	AGATGCACGACGGTTTGCT
Nfkb1	SYBR Green	Mouse	CCACTGTCAACAGATGGCCC	TGGGCCTTCACACACATAGC
Tnip	SYBR Green	Mouse	AGCTGTCACCACCGACATC	GGTAGCAGGATGTACCTGGAC
Tnf-a	SYBR Green	Mouse	TGGCCTCCCTCTCATCAGTT	CTTGGTGGTTTGCTACGACG
Vcam1	SYBR Green	Mouse	TTGGGAGCCTCAACGGTACT	GCAATCGTTTTGTATTCAGGGGA
